# Early life treatment with *Lacticaseibacillus rhamnosus* strains drives reduced enteric methane emissions in dairy heifers

**DOI:** 10.1186/s40104-026-01375-1

**Published:** 2026-04-07

**Authors:** Laureen Crouzet, William Kelly, Catherine Andrews, Priya Soni, Brian Tong, Rebecca Tavendale, Krsana Rajasekaran, Kathy Wieliczko, Kay Pilkington, Rachel Kaminsky, Fiona Matiya, German Molano, Edgar Sandoval, Brenna Dobson-Hill, Peter Reid, Stefan Muetzel, Ajmal Khan, Paul Maclean, Hamish Doohan, Tracey Burgess-Smith, Norton Atkins, Shalome Bassett, James Dekker, Jeremy Hill, Emma Bermingham, Graeme Attwood

**Affiliations:** 1 Rumen Microbiology, AgResearch Ltd, Grasslands Research Centre, Palmerston North, New Zealand; 2 Animal Nutrition and Physiology, AgResearch Ltd, Grasslands Research Centre, Palmerston North, New Zealand; 3 Statistics, AgResearch Ltd, Grasslands Research Centre, Palmerston North, New Zealand; 4 Massey University Dairy Farm 4, Palmerston North, New Zealand; 5 Fonterra Ltd, Fonterra Research and Development Centre, Palmerston North, New Zealand; 6 Riddet Institute, Massey University, Palmerston North, New Zealand; 7 Sustainable Nutrition Initiative, Massey University, Palmerston North, New Zealand

**Keywords:** Enteric methane, Greenhouse gas, Lactic acid bacteria, Rumen, Ruminant

## Abstract

**Background:**

Methane emissions from enteric fermentation in ruminant livestock make up 27% of anthropogenic methane emissions.

**Results:**

Screening > 1,700 lactic acid bacteria identified *Lacticaseibacillus rhamnosus* FNZ118 (Kowbucha™ FNZ118) and *L. rhamnosus* FNZ142, (Kowbucha™ FNZ142) as capable of inhibiting rumen methanogens and methane production in vitro. FNZ118 or FNZ142 fed daily to Friesian heifer calves from birth to 14 weeks substantially lowered methane production through the first year of life compared to control animals. These strains also decreased feed intake and reduced ruminal metabolite concentrations without affecting animal live weight, suggesting an improvement in feed conversion efficiency. The observed effects did not cause major changes in the structure of the rumen microbiome.

**Conclusions:**

These findings demonstrate that early life provision of specific *L. rhamnosus* strains lower CH_4_ production and have potential for the mitigation of enteric greenhouse gas emissions from growing dairy cattle.

**Supplementary Information:**

The online version contains supplementary material available at 10.1186/s40104-026-01375-1.

## Background

Methane (CH_4_) is a greenhouse gas with a 100-year global warming potential nearly 28 times greater than carbon dioxide (CO_2_) [1]. Atmospheric CH_4_ has a relatively short lifetime of 11.8 years [1] compared to CO_2_ which persists for centuries, meaning that reducing CH_4_ emissions has the potential to more quickly impact global warming than reducing CO_2_ emissions. The need to reduce global CH_4_ levels has led to several key initiatives, most recently the Global Methane Pledge launched at COP26, which aims to reduce global anthropogenic CH_4_ emissions by 30 percent from 2020 levels by 2030 [2].

Approximately 27% of CH_4_ from anthropogenic sources comes from enteric fermentation in ruminant livestock [3]. However, ruminants are important sources of nutrition globally, including in developing nations where an increase in ruminant-sourced foods is required to support nutritionally balanced diets. Therefore, reductions in ruminant CH_4_ emissions need to be achieved while at least maintaining, and preferably enhancing, their productivity [4]. As such, global CH_4_ reduction targets are required to be consistent with the United Nations’ Sustainable Development Goal 2 of ending hunger, achieving food security and improved nutrition and promoting sustainable agriculture [5].

Gradual reductions in CH_4_ emission intensity (g of CH_4_ per unit of animal product) are occurring over time as animal production systems become more efficient. However, the current rate of decrease is not enough to meet total emissions reduction targets. Mitigations that decrease CH_4_ yield (g CH_4_/kg feed eaten) and total CH_4_ emissions (g CH_4_/d) from ruminant animals are therefore urgently needed [6]. On-farm solutions also need to be easily implemented across a wide range of farming systems including intensive- and pasture-based operations, as well as “small holder” farms within developing nations. Several different mitigation approaches are being investigated to target CH_4_ producing microorganisms (methanogens) resident in the rumen, including the use of specific methanogen inhibitors such as 3-nitrooxypropanol (3-NOP), certain seaweeds, anti-methanogen vaccines and animal breeding [7–11]. However, on-farm adoption is a major challenge to the implementation of CH_4_-mitigating tools, making it essential that any new technologies are easy to administer, low cost, and provide additional benefits to the farmer, such as improved production or enhanced animal health [8]. Additionally, genetic selection for low CH_4_ emitters runs the risk of selecting for smaller animals with low digestibility of organic matter and fibre [12]. Therefore, while solutions such as 3-NOP, red seaweed and other nutritional strategies, have been shown to reduce CH_4_ emissions from ruminant livestock by as much as 30% [13, 14], these alone will not be sufficient to meet the required targets [4], owing in part to the concurrent reductions in animal productivity observed with some of these interventions [15]. Modification of the rumen microbiome to improve animal production has been achieved through various means [16], but directing fermentation towards lower CH_4_ production has proven challenging. The diverse communities of microorganisms that form the rumen microbiome have considerable overlap in their functions, helping to ensure that the rumen provides a stable but flexible microbial environment to cope with changes in forage types and seasonal feed composition [17–19]. However, this microbial plasticity has limited the success of attempts to alter the composition of the rumen microbiota in adult animals [20].

The gastrointestinal (GI) tract of ruminants is central to their productivity, influencing nutrient utilisation, health, and overall performance. Animal production has been enhanced and better economic outcomes have been achieved through optimising GI tract function via nutritional management and health interventions. Ruminants have an immature GI tract at birth [10] and become colonised with a succession of microbes which culminates in a mature rumen microbiome after weaning is complete [21]. However, the period before weaning is crucial for gut and immune system development, being a critical window for establishment of interactions between the microbiome, host gut epithelial cells, and the immune system. This period also provides an opportunity to shape the developing gut to have long term effects on its microbial ecosystem and its physiology [19, 22]. Indeed, manipulation of the developing GI tract microbiome in early life has been proposed as a strategy to influence rumen function and improve fermentation and productivity [20–23]. Early life interventions can be directed to multiple purposes and, given that calf rearing is common across all dairy farming systems, may be a generally applicable way in which CH_4_ mitigation technologies can be delivered to dairy cattle.

Lactic acid bacteria (LAB) are natural inhabitants of the mammalian GI tract and are used on-farm as direct-fed microbials (DFMs) and as silage inoculants. The use of LAB or their metabolites has been proposed either alone or in combination with other probiotics and feed additives (e.g. yeasts [24]), to alter fermentation in the rumen, thus reducing CH_4_ production [25]. It has also been proposed that LAB and/or their metabolites may directly inhibit rumen methanogens or bacteria that produce substrates for CH_4_ production, such as H_2_ or methyl-containing compounds. Many LAB also produce secreted peptides such as bacteriocins which have been shown to reduce CH_4_ both in vivo and in vitro [26, 27], as well has having immunomodulatory and anti-microbial effects [28]. However, to date, there has been limited research on the ability of exogenous LAB to influence CH_4_ emissions from ruminant animals [9, 29, 30].

Here we report the screening of over 1,700 strains of LAB for their ability to inhibit rumen methanogens and reduce CH_4_ emissions in vitro. Based on the in vitro results, *Lacticaseibacillus rhamnosus* FNZ118 and *L. rhamnosus* FNZ142 were selected for use in an early-life intervention study in dairy calves. New-born Friesian heifer calves received a single daily dose from birth until weaning at 14 weeks of either a control (placebo), FNZ118 or FNZ142 and were monitored for the first 12 months of life to assess the effect of treatment on CH_4_ production and liveweight gain, a key metric used to assess animal productivity for both dairy and beef production [31].

## Methods

### LAB strain selection and preparation of cell extracts

A wide range of species and strains were selected from the Fonterra culture collection and cell extracts were tested for their ability to inhibit indicator methanogen strains representing four representative groups of archaea present in the rumen of dairy cattle: *Methanobrevibacter boviskoreani* JH1 DSM 25824 (‘JH1’) [32], *Methanosphaera* sp. WGK6 (‘WGK6'), *Methanobrevibacter ruminantium* M1 DSM 1093 (‘M1’) and *Methanobrevibacter gottschalkii* D5 (‘D5’) [33] (Fig. S1). LAB strains were revived from −80 °C storage by plating onto De Man-Rogosa-Sharpe (MRS) agar (Merck, NZ Ltd.) (48 h, 37 °C) to obtain a single colony which was re-streaked (MRS, 48 h, 37 °C). A single colony was inoculated into MRS broth (37 °C, 48 h) and 1 mL of the resulting culture was used to inoculate 16 mL of MRS broth containing a very low level of nisin (1 ng/mL final concentration) to induce bacteriocin production. Cultures were incubated overnight at 37 °C and treated as described by Gaspar et al. [34], with the following modifications: the pH of the culture was adjusted to ~ 6.8 with 6 mol/L NaOH, then 0.3 mL of catalase (2 mg/L) was added and the culture incubated for 30 min at 37 °C, followed by an incubation at 70 °C for 45 min. Cultures were centrifuged (20 min, 12,000 × *g*, 4 °C), the supernatant decanted, and the cell pellet resuspended in 8 mL 0.9% NaCl, pH 2. The pH of the resuspended pellet was checked and, if necessary, adjusted to pH 2 with 1 mol/L HCl. Cells were incubated for 2 h at 4 °C with slow agitation on a shaking platform then centrifuged (20 min, 12,000 × *g*, 4 °C) and the supernatant collected into a fresh tube. The pH of the supernatant was adjusted to pH 6.8 with 1 mol/L NaOH and filtered through a sterile filter (Millex-GP 0.22 µm, 25 mm diameter, Millipore, Merck, Sigma-Aldrich NZ) into a sterile, N_2_-flushed, Hungate tube under sterile conditions. These filtered supernatants (cell extracts) were stored frozen (−20 °C) until use.

### Methanogen growth and microtitre plate growth inhibition assays

To identify potential candidate LAB strains with anti-methanogen activities, microtitre plate-based methanogen growth inhibition bioassays were used, employing model methanogen strains. *Methanobrevibacter boviskoreani* JH1 [32] was grown in Balch tubes (Anaerobic tube, 18 × 150 mm, butyl rubber septum stopper, aluminium crimps, Bellco Glass, Vineland, NJ, USA) containing 9 mL BY medium [35] supplemented with (final concentrations) 60 mmol/L sodium formate, 200 mmol/L ethanol, 0.1 mL of Vitamin Solution (1 ×) and 0.1 mL of Coenzyme M Solution (10 µmol/L) by syringe using anaerobic techniques, and incubated at 39 °C without shaking until visible turbidity appeared after 3 to 5 d (OD_600_ 0.8–1.0). Over-pressure in the culture tubes was released prior to removing the inoculum.

*Methanosphaera* make up around 8% of rumen methanogens [17] and are generally H_2_-dependent methylotrophs, using H_2_ to reduce methanol to CH_4_. *Methanosphaera* sp. WGK6 is a H_2_-utilising methylotrophic methanogen isolated from the gut of a kangaroo in Australia, but it is also able to use ethanol as a source of reducing power to reduce methanol to CH_4_ [36] which allows WGK6 to grow on ethanol without the need for an over-pressure of H_2_. A high through-put plate assay was developed using stainless steel gas cannisters able to be pressurized (H_2_ + CO_2_; 80:20). Cultures were grown in Balch tubes in 9 mL BRN-RF10 medium [37] supplemented with (final concentrations) 60 mmol/L sodium formate, 1% methanol, 0.1 mL of Vitamin Solution (1 ×) and 0.1 mL of Coenzyme M Solution (10 µmol/L) using anaerobic techniques with 180 kPa over-pressure of H_2_ + CO_2_ (80:20, BOC Gases NZ) and incubated at 39 °C without shaking until visible turbidity appeared after 3 to 5 d (OD_600_ 0.8–1). Over-pressure in the culture tubes was released prior to removing the inoculum.

*Methanobrevibacter ruminantium* M1 and *Methanobrevibacter gottschalkii* D5 were grown as described for WGK6 above, with the exception that these strains required BY medium for growth. Cultures for the assays were grown in Balch tubes in 9 mL BY medium supplemented with (final concentrations) 60 mmol/L sodium formate, 0.1 mL of Vitamin Solution (1 ×) and 0.1 mL of Coenzyme M Solution (10 µmol/L) using anaerobic techniques with 180 kPa over-pressure of H_2_ + CO_2_ (80:20, BOC Gases NZ). Cultures incubated at 39 °C without shaking until visible turbidity appeared after 3 to 5 d were used for inoculation of the microtitre plate assays.

For the *M. boviskoreani* JH1 growth inhibition assay, cell extracts stored frozen under anaerobic conditions in Hungate tubes were thawed at room temperature. All assay components, except the JH1 inoculum, were added via CO_2_-flushed syringes and needles to 3.75 mL BY + formate medium in sterile 7.5 mL Hungate tubes in the proportions indicated in Table S1. Each tube was then inoculated with freshly grown JH1 culture, incubated for 1 h at 39 °C, then moved inside an anaerobic chamber (98% CO_2_–2% H_2_ atmosphere; Coy Laboratory Products, USA) and dispensed into wells of 96 well plates. Assay plates were placed into an AnaeroPack 2.5 L Rectangular Jar with an MCG Anaeropack-Anaero (Ngaio Diagnostics, Nelson, NZ), the lid sealed, and the jar removed from the anaerobic chamber and incubated at 39 °C. Plates were observed daily until the *M. boviskoreani* JH1 control wells showed visible turbidity (usually within 5 to 6 d). The optical density of each well was then recorded at 595 nm (OD_595_) after 5 s shaking in a Multiskan FC Microplate Photometer (Thermo Scientific, Auckland, NZ). Absorbance readings of the media control wells were subtracted as background, and the % inhibition of *M. boviskoreani* JH1 growth relative to the JH1 positive growth control wells was calculated.

For the *Methanosphaera* sp. WGK6 assay, all assay components, except the WGK6 inoculum, were added via CO_2_-flushed syringes and needles to 3.75 mL BRN-RF10 medium in Hungate tubes supplemented with 1% methanol (247 mmol/L, final concentration) as described in Table S2. Tubes were then moved into an anaerobic chamber with the inoculum tube. Medium was dispensed into the plates then the inoculum added to the appropriate wells. Up to four plates were placed into a stainless-steel gas cannister laid horizontally. Two anaerobic sachets (MCG Anaeropack-Anaero, Ngaio Diagnostics, Nelson, NZ) were added, then the cannister was sealed and removed from the anaerobic chamber, pumped to a pressure 180 kPa with H_2_ + CO_2_ (80:20, BOC Gases NZ) and incubated at 39 °C for 1 week. Cannisters were checked periodically to ensure an over-pressure was maintained, and if necessary, re-pressurised with H_2_ + CO_2_. Following incubation, the contents of each well were resuspended evenly with a multichannel pipettor and the optical density of each well measured at 595 nm (OD_595_) after 5 s shaking in a Multiskan FC Microplate Photometer (Thermo Scientific, Auckland, NZ). Absorbance readings of the media control wells were subtracted as background, and the % inhibition of *Methanosphaera* sp. WGK6 growth relative to the WGK6 positive growth control wells calculated.

Cultures for the *M. ruminantium* M1 and *M. gottschalkii* D5 assays were prepared as described above. Over-pressure in the tubes was released prior to removing the inoculum. Assay components were added to 3.5 mL of sterile BY medium in a 7.5 mL Hungate tube via CO_2_-flushed syringes and needles as described in Table S3. Each tube was then inoculated with freshly grown culture, incubated for 1 h at 39 °C, then moved inside the anaerobic chamber and dispensed into the wells of a 96-well plate. Plates were sealed and incubated in stainless steel gas cannisters under 180 kPa over-pressure of H_2_ + CO_2_, and their OD_595_ measurements recorded as described for the *Methanosphaera* sp. WGK6 assay. The OD_595_ readings of the BY media control wells were subtracted as background, and the % inhibition of the growth of the *M. ruminantium* M1 or *M. gottschalkii* D5 relative to the positive growth control wells were calculated.

### Preparation of LAB cultures and filtrates, set up of rumen in vitro (RIV) assays and end product analysis

LAB strains were inoculated into seven Hungate tubes, each containing 5 mL of anaerobic MRS medium (Sigma-Aldrich) and incubated at 39 °C for 16 h (until the cultures reached stationary phase). Cultures were pooled into a 250 mL CO_2_-flushed serum bottle. One mL of the combined culture broths was added to 9 mL of sterile MRS medium and the OD_600_ measured. Further aliquots (0.5 mL) of the culture mix were inoculated in triplicate into 4.5 mL of sterile anaerobic buffer, serially diluted tenfold under CO_2_ and plated onto MRS plates to determine the number of colony forming units (CFU/mL) of original culture. Half of the remaining culture was used for one set of rumen in vitro fermentations (designated as culture) and the other half was filtered (Millipore 0.22 μm pore size) and the filtrate was placed into a new sterile anaerobic serum bottle (designated as filtrate). Anaerobic phosphate buffer (0.46 mol/L K_2_HPO_4_; 0.54 mol/L KH_2_PO_4_, pH 7) was used as a no treatment control (Buffer), while a *L. bulgaricus* overnight culture filtrate (LBF) which has no inhibitory effects in RIVs, but which accounts for end products from LAB cultures (mainly lactate) which contribute to RIV fermentation was used as an additional control.

For inoculation of the rumen in vitro fermentation vessels, fresh rumen contents were collected from six rumen-fistulated Friesian cows. After squeezing through one layer of cheesecloth, the resulting rumen fluids from two animals were combined (approx. 150 mL rumen fluid) giving three biological replicates. Aliquots (12.5 mL) of the mixed rumen fluid were added to 0.5 mg dried grass and 36.5 mL of anaerobic phosphate buffer in a 250 mL serum bottle. The treatments (1 mL) of either Buffer, test culture, supernatant or cell extract were added before closing the serum bottles with butyl rubber stoppers, giving a final fermentation volume of 50 mL containing 25% rumen fluid (v/v). Gas production and CH_4_ content were measured continuously for 24 h using an automated incubation system [38].

Samples were collected from bottles for VFA analysis. At each time point (2, 6, 12 and 24 h), 3 mL aliquots were collected, and their pH measured. Samples (1.8 mL) of these aliquots were used for VFA and formate + non-VFA (lactate, succinate) analyses (see below).

### Animal trial design

A simple two treatment (FNZ118, FNZ142) versus control (excipient only) randomised complete block design was used. A statistical power analysis based on standard deviations of CH_4_ emission measurements in young calves indicated 20 animals per group were required to detect a 10%–15% difference in CH_4_ emissions. To allow for exclusion of calves from the trial through health interventions (due to navel infections or scouring which requires antibiotic treatment), a total of 72 new-born female Friesian heifer calves were enrolled into the study over a 3-week period from calves born to the Massey University Dairy 4 dairy cow herd. Calf enrolment was staggered over this period to spread the calf age such that similarly aged animals could be measured sequentially through the cattle CH_4_ measurement chambers at the New Zealand Ruminant Methane Measurement Facility (NZRMMF) at AgResearch Grasslands [39]. CH_4_ measurements were not conducted on the FNZ142 treatment group at 9 months of age as this treatment group had shown no differences compared to the control at the 14-week measurement period. However, subsequent analysis of rumen contents indicated changes in the rumen microbiome similar to those of FNZ118-treated calves therefore FNZ142 was also included in the 12-month measurement.

### Calf enrolment and feeding

The calves were selected for the study from calves born to the Massey University Dairy 4 dairy cow herd. Calf enrolment was staggered over a 3-week period to spread the calf age such that similarly aged animals could be measured sequentially through the cattle CH_4_ measurement chambers at the NZRMMF. Newly born calves were collected from their mothers twice daily and taken to the calf rearing shed at the Dairy 4 farm. The calf rearing shed (21.8 m × 13 m = 283 m^2^) was divided into 18 pens to house calves for 14 weeks. Pens were disinfected before the start of the study and bedded with woodchips. Each pen (approximately 3 m × 4 m = 12 m^2^ minimum; 3 m^2^/calf) was used to house four calves and was equipped with a drinking water supply and a pelleted meal feeder. Calf liveweights (LWTs) were recorded on arrival and then weekly during their time in the calf rearing facility. Calves were assigned at random to three treatment groups (FNZ118 treatment, FNZ142 treatment, or control) over a 3-week period. These assignments were balanced with respect to calf birth weight and sire, to achieve two pens (eight calves) per treatment group per week during the 3-week enrolment. There were 72 calves enrolled in total, with 24 calves housed in 6 pens for each treatment group. Each pen holding 4 calves received a single treatment type. Vaccinations and other animal health treatments given to the calves are shown in Supplementary Text.

The FNZ118, FNZ142 and Control treatments were fed to calves once daily from birth, initially mixed with colostrum, then via Calf Milk Replacer (CMR; Ancalf™, NZAgBiz; 150 g/L mixed with tap water at ~ 37 °C). Within the first 12 h of entering the calf rearing shed, each calf was offered 2–3 L of warm colostrum, twice daily. The morning colostrum feed contained FNZ118, FNZ142 or control treatment. Freeze-dried FNZ118 or FNZ142 was supplied by Fonterra as 3 g (1 calf dose, 5 × 10^10^ CFU FNZ118 or FNZ142) aliquots in sealed foil sachets and stored at −20 °C until use. The Control dose (3 g maltodextrin/calf/d) was the excipient used for blending of the freeze-dried lactic acid bacterial powder. The FNZ118, FNZ142 and Control treatments were separately resuspended in small amounts of warm water (~ 37 °C) prior to adding to the more viscous colostrum, to aid mixing and dispersal within the colostrum. Calves were fed colostrum containing FNZ118, FNZ142 or Control in the morning feed for 2 to 4 d after birth (and colostrum only in the afternoon) and were transitioned gradually onto CMR from d 2 after birth. Calves received a full CMR diet by the end of the fourth day of feeding after birth. After colostrum feeding ended, calves were fed 6 L CMR daily, divided into 3 L morning and 3 L afternoon feeds with treatments only added to the morning feeds. FNZ118, FNZ142 and Control treatments were added to prewarmed CMR and mixed until the freeze-dried material was evenly distributed throughout the milk. From week two after birth, calves were also offered solid feed in the form of a pelleted calf feed (Denver Feeds, Palmerston North) containing 20% fibre source (lucerne and soy hulls) and a non-ionophore coccidiostat (Table S4). No hay was offered to calves during the first 6 weeks after birth to minimise variation in solid feed consumption between calves, and the possible impact of hay consumption on rumen development and calf CH_4_ emissions. However, meadow hay and chaffed meadow hay (cut to ~ 75 mm) were offered to the calves after their first CH_4_ measurements at 6 weeks of age to help stimulate salivary secretions and stabilise rumen pH. The intake of pelleted feed and hay was measured at the pen level (4 animals). Calves were weaned off most of their milk starting from the end of week 10 after birth so that by the end of week 11 they were receiving only 0.5 L of CMR in the morning feed containing either the FNZ118, FNZ142 or Control dose until 14 weeks of age. When the milk and treatment feeding stopped, the calves were released onto pasture so that by the end of the treatments each calf had been dosed for 14 weeks.

### Animal CH_4_ emission measurements and sampling

**Fig. 1:**
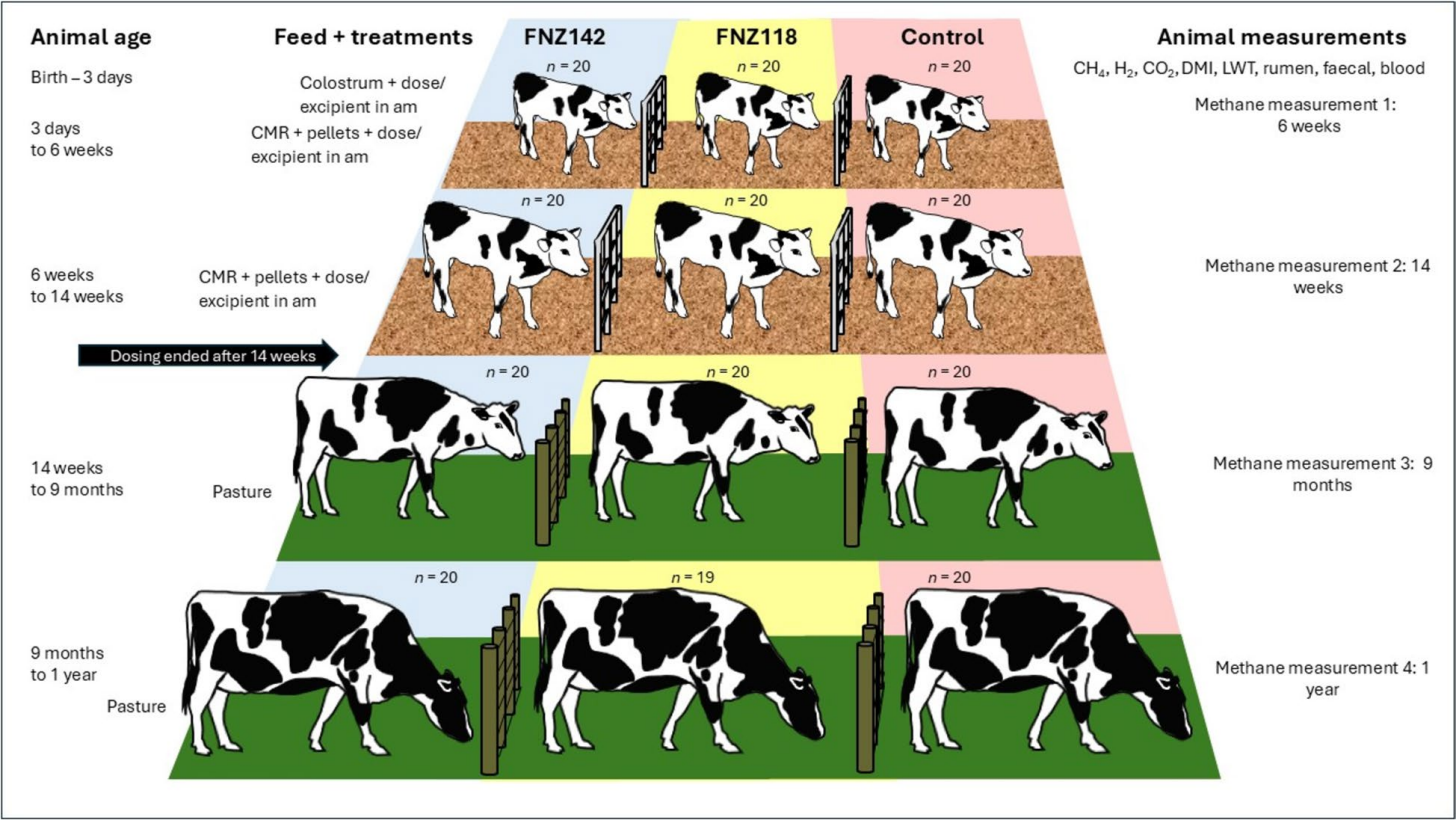
Experimental design. 60 Female Friesian calves (20 per treatment group, selected from 72 enrolled at birth) were measured for CH_4_ emissions at 6 weeks (pre-weaning), 14 weeks (post weaning), 9 months and 1 year of age. Calves either received a daily dose of 3 g of freeze-dried FNZ118 or FNZ142 (5 × 10^10^ CFU) or Control (maltodextrin excipient) from birth until the completion of CH_4_ measurements at 14 weeks of age. At week 15, calves were transitioned off pellets to pasture over a 3-week period and continued to graze pasture until 1 year of age

A total of 60 (20 per treatment group) calves were selected from the originally enrolled 72 calves for CH_4_ measurements based on their previous health records and whether they had received anti-inflammatory drugs and/or antibiotics for health reasons (Fig. 1). CH_4_ measurements were carried out at 6 weeks (pre-weaning), 14 weeks (post-weaning), 9 months (except for the FNZ142 group) and 1 year of age in four cattle respiration chambers at the NZRMMF, 4 animals at a time for 2 d each. Intakes of milk and solid feed (calves) and pasture (heifers at 9 months and 1 year of age) were measured in each round of CH_4_ measurements, and samples of the feed were dried to calculate the dry matter intake (DMI) per animal per day and submitted to the Massey University Nutrition Laboratory for compositional analysis. Respiration chamber measurements of CH_4_, H_2_, and CO_2_ were reported as emissions (g/d) or as yields (g/kg DMI/d). Calves at 6 weeks of age (4 per trip) were transported from the calf rearing shed at Massey University Dairy 4 to the AgResearch NZRMMF in a covered trailer and were housed in pens with wood chip bedding. On arrival they were given their afternoon allowance of milk (3 L, pre-weaning) and had fresh water available. The following morning the calves were moved into individual cattle respiration chambers where they received their respective treatments (3 L CMR/calf with FNZ118, FNZ142 or Control) and had pelleted feed and water available ad libitum. All feed intake was recorded individually. Calves continued to receive their treatments daily in the morning and solid pelleted feed ad libitum while in the chambers for two days of CH_4_ measurements. Calf feeding and cleaning of the pens occurred twice daily. On the morning of the third day, calves were removed from the chambers to a pen and fed their morning allowance of milk with treatments added. Two hours after this morning feeding, calves were sampled for rumen contents (via stomach tubing) and faecal material (via digital collection) which were used immediately for pH measurements with the remainder flash frozen in liquid N_2_ and stored at −80 ^◦^C for subsequent VFA, non-VFA, and microbial community analyses. Blood samples (10 mL) were collected from the jugular vein of each animal into serum vacutainer tubes (BD Vacutainer, Fisher Scientific, NZ). Blood samples were centrifuged at 2,000 × *g* for 20 min at 10 ^◦^C and the supernatant (serum) was stored frozen at −20 °C.

After each set of 4 animals completed their CH_4_ measurements and samplings they were returned to Massey University Dairy 4 where they continued to receive their treatments via the morning milk feed and pelleted calf feed and additionally meadow hay was offered ad libitum until 12 weeks of age. From 12 to 14 weeks of age, meadow hay was replaced by chaffed meadow hay ad libitum and when the calves were transported back to the NZRMMF respiration chambers for the post-weaning measurement at 14 weeks of age, chaffed meadow hay was offered at ~ 10% (500 g/d) of the weight of pelleted calf feed offered (5 kg) per animal, along with the 0.5 L of CMR in the morning feed containing either the FNZ118, FNZ142 or Control dose. Samples of rumen contents, faecal material and blood were collected from animals after exiting the respiration chambers as described above. After each set of 4 calves completed their CH_4_ measurements and samplings at 14 weeks of age, they were transported to the Massey University Sheep, Beef and Cattle Research Unit where they were managed in different paddocks for adaptation to pasture feeding. The pasture was mainly perennial ryegrass with some white clover and calf pellets were initially available at their previous daily intake and the quantity of pellets offered was reduced over a 3-week period to encourage the transition to an all pasture diet (33% reduction in pellet allowance per week). The animals (now weaner heifers) were maintained on pasture until 9 months of age when the Control and FNZ118 groups were transported via a commercial stock trucking company back to the NZRMMF for their third round of CH_4_ measurements. CH_4_ measurements were not conducted on the FNZ142 treatment group at 9 months of age as they showed no differences compared to the Control at the 14-week measurement period. The heifers were selected at random and adapted to cut pasture in pens for 5 d. The pasture was a mixed sward of ryegrass and clover harvested daily in the morning from the AgResearch Aorangi Farm and which was similar in composition to the pasture at the Massey University farm. The cut pasture was transported to the NZRMMF and half was fed fresh to the animals as their afternoon feed, and the remaining grass was stored at 4 °C in a chiller and used the following day for the morning feed. The heifers were moved to individual cattle crates for an additional 2 d where they continued to receive cut pasture and then into cattle respiration chambers for 2 d. The feed offered and refused while in crates and in respiration chambers was recorded to calculate individual intakes. The feeding of heifers and cleaning of the pens occurred twice daily. Samples of the feed were collected and dried at 105 °C for 24 h to calculate the DMI per heifer per day. Sub-samples of the feed dried at 65 °C for 48 h were also submitted to the Massey University Nutrition Laboratory for compositional analysis (Table S4). After completion of the CH_4_ measurements and samplings (as described for the calf samplings) at 9 months of age, all the animals were returned to Massey University and grazed at the Keebles Farm. At 1 year of age all the animals (including the FNZ142 group) were returned to the NZRMMF for a fourth round of CH_4_ measurements and samplings which were conducted as for the 9 month measurements described above. Heifer LWTs were recorded monthly while grazing at Massey University, and pasture samples were collected and stored for subsequent analyses. The FNZ142 animals were included in the 12-month measurement as analysis of their rumen contents collected at 9 months indicated changes in the rumen microbiome similar to those of FNZ118-treated calves at 9 months of age.

### VFA and non-VFA analyses

Samples of rumen contents were thawed at room temperature and centrifuged at 21,000 × *g* for 10 min at 4 °C. An aliquot (0.9 mL) of supernatant was removed and added to 0.1 mL of internal standard (20 mmol/L 2-ethylbutyrate in 20% phosphoric acid), mixed and frozen at −20 °C until analysis. After thawing and re-centrifugation at 21,000 × *g* for 10 min at 4 °C, 0.2 mL of the supernatant was collected for derivatization for non-VFA analysis using gas chromatography, while the remainder of the sample (0.8 mL) was analysed directly via gas chromatography [40], using a gas chromatograph (Model 6869, Hewlett-Packard, Montreal, QC, Canada) equipped with an auto-sampler, fitted with a Zebron ZB-FFAP 30.0 m × 0.53 mm I.D. × 1 μm film column (Phenomenex, Torrance, CA, USA) and a flame ionization detector set at 265 °C. The derivatization procedure converted the acids to tertiary butyldimethylsilyl derivatives prior to separation by GC [41].

### DNA extractions and microbial community analyses

Frozen samples of rumen contents and faeces were thawed, and the DNA extracted using a bead-beating/phenol chloroform method [42]. The DNA was used in PCR reactions to generate 16S ribosomal RNA gene amplicons with barcoded sequencing primers specific for bacteria (Ba9F/Ba515Rmod1) and archaea (Ar915aF/Ar1386R) targeting the variable regions V1–V3 and V6–V8 respectively [43]. Amplicons were purified, normalised, pooled, and sequenced via an Illumina MiSeq sequencer at Massey Genome Services. Sequencing results were quality controlled and filtered and the filtered sequences were analysed via QIIME pipeline (V1.9.1) using the Silva database (version 132; 99% OTUs) with rumen-specific 16S ribosomal RNA gene sequences. Operational taxonomic units (OTUs) were picked at 97% and tabulated.

### Quantitative PCR (qPCR) analysis of rumen methanogen abundance

Methanogen abundances within rumen and faecal samples were assessed by enumerating 16S rRNA gene copies from extracted total DNA using qPCR with methanogen group-specific primers (Table S5) for *Methanobrevibacter gottschalkii*, *Methanobrevibacter* spp., Methanomassiliicoccales spp., total rumen methanogens and primers for total rumen bacteria. The qPCRs were carried out using a Rotor-Gene 6000 real-time Rotary Analyzer (Corbett Life Science, Concorde, NSW, Australia) and amplicon detection was by SYBR Green I fluorescence (for archaea; LightCycler 480 DNA SYBR Green I Master for Ar Kit Cat # 04707516001 and for bacteria LightCycler FastStart DNA Master SYBR Green I Kit, Roche Diagnostics NZ Ltd, Auckland, NZ [44]. The amplicon copy numbers in the standards were calculated from the amplicon concentration determined by the Quant-iT kit (Invitrogen NZ Ltd., Thermo-Fischer Scientific, Auckland, NZ). Serial dilutions of external DNA standards from 10^9^ to 10^5^ copies per reaction were prepared and used with each qPCR run to obtain standard curves which were used to calculate the number of archaeal and bacterial 16S rRNA gene copies in the extracted DNA from the rumen and faecal samples. Differences in 16S rRNA gene abundances were analysed by using LinRegPCR V12 [45]. Gene copy numbers were determined by normalizing the qPCR measurements to the standard curve using Rotor-Gene 6000 Series Software (V 1.7).

### Statistical analyses

The pure culture methanogen inhibition RIV data, animal emission and VFA data were loaded into R version 4.3.0 [46] and analysed using Restricted Estimate Maximum Likelihood analysis in lme4 R package version 1.1–34 [47] with log-transformation to stabilise the variance. For the repeated measure analyses, time (sampling time of the study), treatment, and their interaction (treatment × time) were used as fixed effects, and animal was used as the random effect. For the RIV data bottle was used as the random effect. Subsequent statistics such as treatment means, and 95% confidence intervals or SEM were calculated using the *predictmeans* R package version 1.0.8 [48]. The microbiome data was loaded into R version 4.3.0 [46] and separated by superkingdom and environment. Rarefaction curves were generated using the “vegan” R package version 2.6–4 [49]. Proportions were calculated for each sample by dividing the sequencing read count of each OTU by the total sequencing read counts for the sample. Principal coordinate analysis (PCoA) was performed on the proportions with the “APE” package version 5.7–1 [50] on Bray–Curtis distances [51] calculated using the “vegan” R package version 2.6–4 [49]. Confidence ellipses (95%) were calculated on the standard errors of the ordinations using the “ordiellipse” function. Diversity estimates and ANOSIM [52] was also performed using the “vegan” R package with the default settings (including Bray–Curtis distance calculations). The microbiome data was converted into a phyloseq object using the phyloseq package version 1.44.0 [53], then an ANCOM-BC analysis was run on the phyloseq object using the ANCOMBC2 function in the ANCOMBC R package version 2.2.2 [54] with the treatment factor being analysed and the control treatment being the first level of the treatment factor.

## Results

### Inhibition of methanogen strains in vitro

To test whether specific LAB strains could inhibit these methanogens, cell extracts from a total of 1,725 strains of LAB were screened for their ability to inhibit the growth of *Methanobrevibacter boviskoreani* JH1 and *Methanosphaera* sp. WGK6 using either an existing methanogen growth inhibition assay (JH1 [32]) or new (WGK6) assay developed in this study (Table 1). The LAB strain extracts demonstrated a range of inhibitory activities against the JH1 and WGK6 methanogen strains, and in some cases stimulated methanogen growth. To test the species spectrum of inhibitory activities, cell extracts from 200 LAB strains were further screened in growth inhibition assays developed during this study using *M. ruminantium* M1 and *M. gottschalkii* D5. The 200 LAB strains included the 100 best-performing strains from the JH1 and WGK6 assays, as well as an additional 100 strains, representing 16 LAB species, randomly selected from the remaining 1,625 LAB strains (Table 1).

**Table 1:** Summary of LAB strains screened for their ability to inhibit or stimulate the growth of methanogen type strains in vitro

Methanogen strain	LAB strains screened	Number of LAB strains showing inhibition:				Stimulation of growth
		0%	10%–50%	50%–80%	> 80%	
*M. boviskoreani* JH1	1,725	506	480	37	83	619
*Methanosphaera* sp. WGK6	1,725	610	325	71	17	702
*M. ruminantium* M1	200	67	46	1	0	86
*M. gottschalkii* D5	200	67	31	0	0	102

In general, the inhibitory activities observed in the methanogen screening assays were greatest against *M. boviskoreani* JH1 and *Methanosphaera* sp. WGK6, while the majority of the 200 LAB strains tested showed little or no inhibition of the *M. ruminantium* M1 and *M. gottschalkii* D5 methanogens. This is possibly due to the lower number of LAB strains that were tested against M1 and D5, and screening the remaining 1,525 LAB strains may identify more candidates active against M1 and D5.

*Lacticaseibacillus rhamnosus* HN001 was previously shown to decrease *Methanobrevibacter* in the caecal microbiome of piglets [55], so we were particularly interested in how *L. rhamnosus* strains performed in these assays. Of the 1,725 LAB strains screened, 94 strains were classified as *L. rhamnosus* and of these, 62 (~ 66%) showed less than 20% inhibition of WGK6, 81 (~ 86%) showed less than 50% inhibition, and only 3 strains (~ 3%) showed ~ 80% inhibition or more. Together, this indicates that methanogen inhibition in these assays is a strain-specific effect. The top performing *L. rhamnosus* strains, *L. rhamnosus* FNZ118 and *L. rhamnosus* FNZ142, showed very strong inhibition of the indicator methylotrophic methanogen *Methanosphaera* sp. WGK6, weaker inhibition of the indicator hydrogenotrophic methanogen *M. boviskoreani* JH1, and very weak or no inhibition of *M. ruminantium* M1, or *M. gottschalkii* D5 (Table 2).

**Table 2:** Inhibition of rumen methanogen strains in vitro by cell extracts of FNZ118 and FNZ142

Strain	% of control growth			
	JH1	WGK6	M1	D5
*L. rhamnosus* FNZ118	98.7 ± 0.6	21.2 ± 4.6^**^	109.6 ± 4.8	102.5 ± 5.0
*L. rhamnosus* FNZ142	76.3 ± 1.3^**^	0.4 ± 0.6^**^	101.0 ± 5.6	96.4 ± 4.9

Numbers represent % of methanogen growth with the extract added compared to addition of buffer only control. Numbers greater than 100% indicate a stimulatory effect of the extract. ^**^P < 0.01

Based on these results of the methanogen screening assays, the *L. rhamnosus* strains FNZ118 and FNZ142, were further tested in rumen in vitro assays.

### Inhibition of CH_4_ production in vitro

A rumen in vitro (RIV) model was used for testing the effects of the top candidate *L. rhamnosus* strains from the plate-based in vitro screening assays on a simulated rumen microbial community [38]. The *L. rhamnosus* FNZ118 culture and filtrate showed no significant effect on total gas produced in the RIV assays (Table 3). However, FNZ118 culture produced a significant decrease in total CH_4_ at 2 and 6 h in two independent RIV runs. This effect was not seen using FNZ118 culture filtrate. There was also no significant effect of FNZ118 culture on the total SCFAs concentrations, or the concentrations of individual SCFAs produced (Table S6). *L. rhamnosus* FNZ142 culture produced a significant decrease in total CH_4_ produced at 2 and 6 h in RIV Run 1, but not in a repeated RIV assay (Table 3). The FNZ142 culture filtrate showed no significant effect on CH_4_ production. The *L. rhamnosus* FNZ142 culture caused a slight decrease in total gas production, although this only reached significance at 6 h and 24 h in RIV Run 1, while the filtrate showed no effect on the total gas produced. There was no significant effect of FNZ142 on total SCFAs concentrations, or the individual SCFA concentrations measured (Table S6).

**Table 3:** Inhibition of rumen in vitro CH_4_ production by culture, or culture filtrates of *L. rhamnosus* FNZ118 or FNZ142

LAB strain	Fraction	RIV run	Total gas				CH_4_			
			2 h	6 h	12 h	20 h	2 h	6 h	12 h	20 h
FNZ118	Culture	1	91.7 ± 1.6	91.4 ± 2.4	94.1 ± 1.6^*^	94.0 ± 1.6^*^	79.5 ± 1.9	78.8 ± 2.1^*^	90.9 ± 1.4^*^	93.2 ± 1.6^*^
	Filtrate	1	101.9 ± 11.5	94.0 ± 3.6	99.8 ± 3.0	98.5 ± 1.7	103.1 ± 0.5	96.2 ± 1.0	102.8 ± 2.0	100.1 ± 1.8
	Culture	2	97.3 ± 1.8	98.8 ± 2.0	99.0 ± 1.4	100.3 ± 1.6	86.0 ± 1.8^*^	92.3 ± 2.0	96.4 ± 1.5	98.6 ± 1.6
FNZ142	Culture	1	94.5 ± 1.2	90.4 ± 0.5^*^	94.0 ± 0.7^*^	93.6 ± 0.6^*^	72.4 ± 0.5	76.4 ± 0.4^*^	88.2 ± 0.3^*^	90.5 ± 0.7^*^
	Filtrate	1	108.5 ± 4.2	93.5 ± 1.3	95.3 ± 1.3	93.7 ± 0.2^*^	100.2 ± 3.8	91.6 ± 1.8	96.0 ± 1.5	94.2 ± 0.1^*^
	Culture	2	103.8 ± 2.4	99.7 ± 1.7	99.2 ± 1.4	99.9 ± 1.4	95.1 ± 3.7	95.9 ± 2.1	97.8 ± 1.3	98.7 ± 1.2

Numbers are either total gas volume (mL/g substrate) or CH_4_ volume (mL/g substrate) as % of *L. bulgaricus* filtrate controls ± SEM. Numbers greater than 100% indicate a stimulatory effect. ^*^Mixed effect model *P*-value < 0.05

The observed reductions in CH_4_ emissions in RIVs by culture samples of *Lacticaseibacillus rhamnosus* FNZ118 (Kowbucha™; FNZ118) and *L. rhamnosus* FNZ142 (Kowbucha™; FNZ142), with minimal impact on total gas or VFA production, led to their selection for in vivo testing in dairy calves.

### Animal trial

An animal trial was conducted to test the efficacy of the FNZ118 and FNZ142 strains in reducing CH_4_ emissions when fed to newly born calves until the animals were weaned at 14 weeks.

**Fig. 2:**
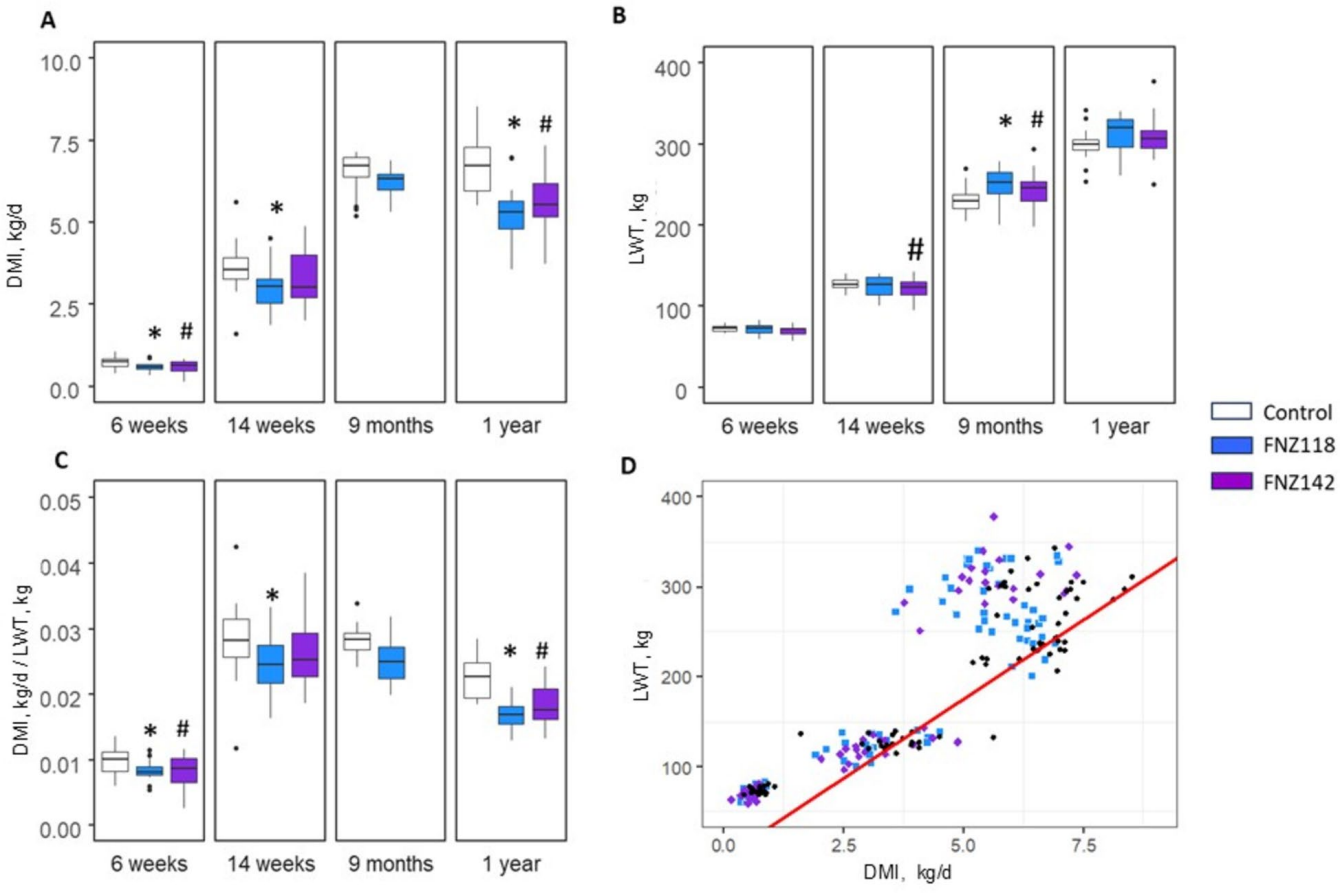
Animal measurements while in respiration chambers. Data are shown as Box plots with median and quartiles. **A** Dry matter intake (DMI). **B** Post-chamber live weight. **C** DMI normalized against animal weight at 6 weeks, 14 weeks, 9 months and 1 year of age. **D** Animal DMI plotted against LWT; the red solid line indicates the New Zealand dairy cattle average of approximately 3% of LWT [56, 57]. White box and whisker plots (or black symbols in Fig. 2D) represent the Control group; blue boxes (or blue symbols in Fig. 2D) represent the FNZ118-treated animals; purple boxes (or purple symbols in Fig. 2D) represent FNZ142-treated animals; DMI and CH_4_ measurements were not conducted on the FNZ142 treatment group at 9 months as this treatment group had shown no differences compared to the Control at the 14-week measurement period. Because subsequent analysis of rumen contents of FNZ142 animals collected at 9 months indicated changes in rumen metabolites similar to those of the FNZ118-treated animals, the FNZ142-treated animals were also included in the 1 year DMI and CH_4_ measurements. ANOVA *P*-value FNZ118 vs. control: ^*^P < 0.05; ANOVA *P*-value FNZ142 vs. control: ^#^*P* < 0.05

The feed intakes of calves were measured at the pen level during calf shed rearing (four animals per pen) and are shown in Table S7. Individual DMI of animals while in chambers at 6 and 14 weeks, and at 9 months and 1 year of age are shown in Fig. 2A and Table 4. By 6 weeks of age, all animals received 6 L of milk per day, in addition to their pelleted feed. However, those animals receiving FNZ118 and FNZ142 demonstrated a reduction in DMI compared to control animals. By 14 weeks, the DMI for the FNZ118 calves remained lower than Controls, while the FNZ142 calves did not differ from Controls. Individual DMI of the weaner animals at 9 months of age did not differ between Control and FNZ118 animals, while at 1 year of age, the yearling heifers in the FNZ118 and FNZ142 treatment groups consumed 22% and 16% less pasture than the Control animals, respectively.

**Table 4:** Dry matter intakes and gas emissions of animals in respiration chambers^1^

Measure	6 weeks			14 weeks			9 months		12 months		
	FNZ118	FNZ142	Control	FNZ118	FNZ142	Control	FNZ118	Control	FNZ118	FNZ142	Control
DMI, kg/d	0.6 ± 0.03^*^	0.56 ± 0.03^*^	0.69 ± 0.03	2.98 ± 0.14^*^	3.21 ± 0.15	3.51 ± 0.17	6.18 ± 0.29	6.5 ± 0.31	5.21 ± 0.25^*^	5.64 ± 0.27^*^	6.74 ± 0.32
CH_4_, g/d	7.04 ± 0.34^*^	8.29 ± 0.4^*^	9.88 ± 0.48	39.42 ± 1.9^*^	45.27 ± 2.18	50.54 ± 2.43	99.81 ± 4.81^*^	116.06 ± 5.59	128.62 ± 6.33^*^	119.06 ± 5.73^*^	156.82 ± 7.55
CH_4_, g/kg DMI	12.06 ± 0.58^*^	15.56 ± 0.74	14.49 ± 0.69	13.34 ± 0.64	14.49 ± 0.69	14.43 ± 0.69	16.2 ± 0.77	17.92 ± 0.85	25.21 ± 1.23	21.28 ± 1.02	23.32 ± 1.11
H_2_, g/d	0.05 ± 0.02	0.07 ± 0.03	0.09 ± 0.03	0.13 ± 0.03	0.12 ± 0.03	0.15 ± 0.03	0.32 ± 0.03	0.38 ± 0.03	0.1 ± 0.03^**^	0.15 ± 0.03^*^	0.27 ± 0.03
H_2_, g/kg DMI	0.08 ± 0.01^*^	0.13 ± 0.01	0.14 ± 0.01	0.04 ± 0.01	0.04 ± 0.01	0.04 ± 0.01	0.06 ± 0.01	0.06 ± 0.01	0.02 ± 0.01	0.03 ± 0.01	0.04 ± 0.01
CO_2_, g/d	1,623.01 ± 42.8^*^	1,647.95 ± 43.46^*^	1,783.45 ± 47.04	2,875.84 ± 75.85^*^	3,166.48 ± 83.51	3,265.9 ± 86.13	4,684.15 ± 123.54	5,059.13 ± 133.43	5,799.21 ± 155.98^*^	5,642.82 ± 148.82^*^	6,525.56 ± 172.1
CO_2_, g/kg DMI	2,798.51 ± 109.82	3,127.15 ± 122.72^*^	2,628.9 ± 103.17	974.85 ± 38.26	1,014.96 ± 39.83	934.38 ± 36.67	760.44 ± 29.84	780.82 ± 30.64	1,130.9 ± 45.54^*^	1,009.73 ± 39.62	970.32 ± 38.08

^1^Numbers are group means over 2-day measurements ± standard error of the mean. Treatment means, and SEM were modelled using the R package *predictmeans*; FNZ118 or FNZ142 treatment vs. Control, ^*^P < 0.05, ^**^P < 0.01. Animal numbers per group: *n* = 20 for FNZ118 and Control groups for 6 weeks, 14 weeks, 9 months; *n* = 20 for Control group at 1 year, *n* = 19 for FNZ118 group at 1 year. *n* = 20 for FNZ142 group. For measurements at week 6, calves consumed 6 L CMR/d (900 g/d milk solids) plus the indicated amount of calf pellets which represented ~ 40% of total intake. For the measurements at week 14 calves consumed 0.5 L CMR/d and were offered pellets (5 kg/d) with 10% of anticipated intake offered as chaffed hay (500 g/d). For measurements at 9 months and 12 months respectively the heifers consumed only pasture (ryegrass-clover mixed sward cut and carried to the animals) in the amounts indicated

The LWT and average daily gains (ADG) data at 6 weeks, 14 weeks, 9 months and 1 year are summarised in Table S8. The modelled data at 6 weeks showed a slight decrease in LWT of FNZ142 animals at 14 weeks of age and an increase in LWT in FNZ118 and FNZ142 animals at 9 months (Fig. 2B). The ADG data showed no differences between Controls and treatment groups at 6 or 14 weeks but increases in ADG in both FNZ118 and FNZ142 animals at 9 months and 1 year. When LWT measured during respiration chamber stays was calculated per unit of DMI, the FNZ142 animals at 6 weeks, the FNZ118 group at 9 months, and both FNZ118 and FNZ142 groups at 1 year, had lower kg DMI/kg LWT (Fig. 2C). The intake per unit of LWT of all of the calves in the current study is also shown relative to the New Zealand average for dairy cattle of approximately 3% of LWT (e.g., [56, 57]; Fig. 2D).

After daily supplementation with FNZ118 and FNZ142 for 14 weeks, the heifers were transitioned off pellets and grazed on pastures. Heifers that had received FNZ118 or FNZ142 showed numerically higher monthly LWTs relative to the control heifers while grazing on pasture, but as intakes could not be measured during this period, these differences were not analysed statistically. Overall, the animals fed with FNZ118 or FNZ142 pre-weaning exhibited lower feed intake without any impact on animal LWT, suggesting a sustained improvement in feed conversion efficiency.

### Calf and heifer CH_4_ emissions measurements

**Fig. 3:**
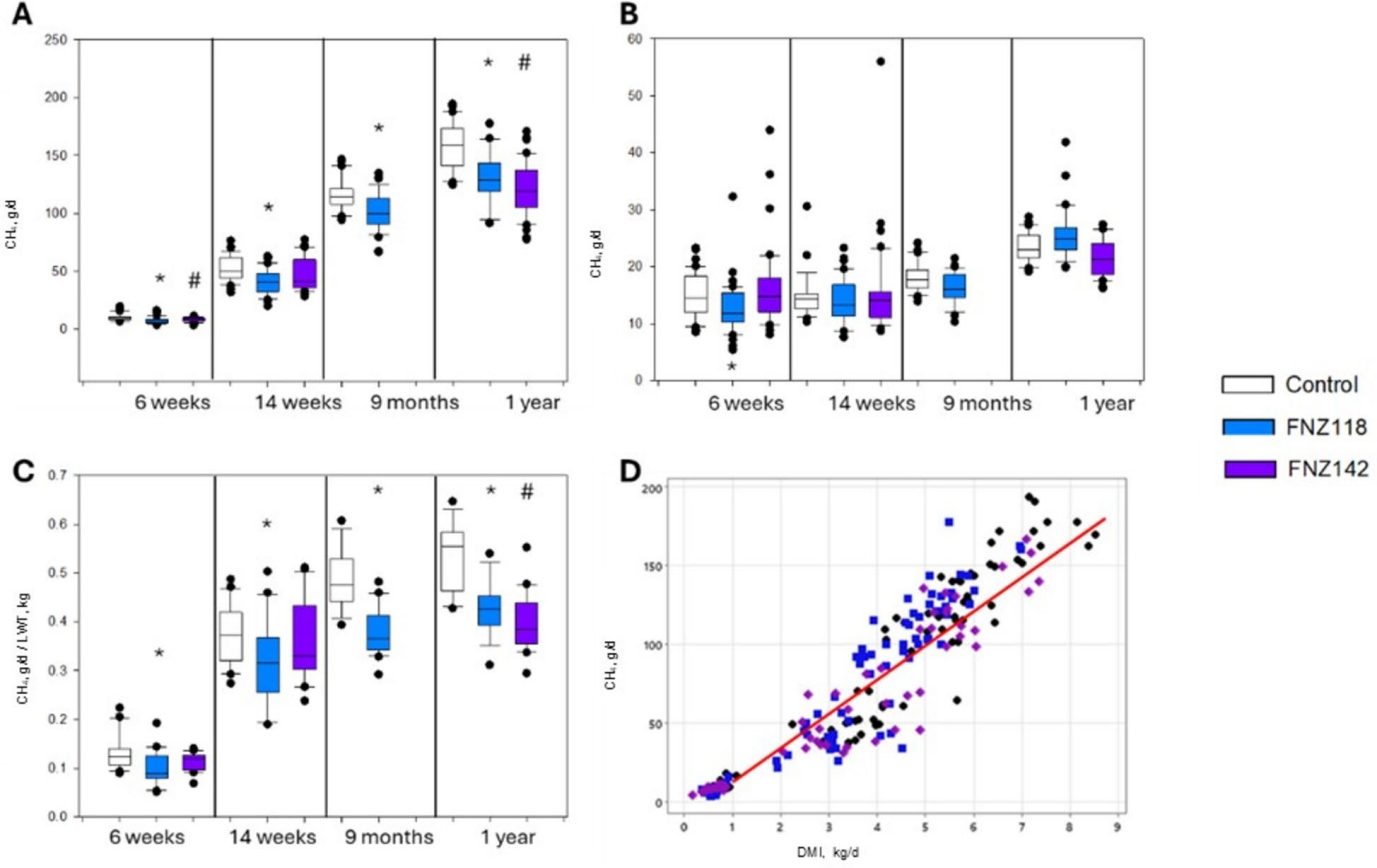
Animal measurements while in respiration chambers. **A–C** CH_4_ emissions (**A**), CH_4_ yield (**B**), and CH_4_ produced per day normalized using animal weight (**C**) at 6 weeks, 14 weeks, 9 months and 1 year of age. Box plot data with median and percentile. **D** CH_4_ emissions plotted against DMI for the experiment and the red solid line indicates the New Zealand average dairy cow CH_4_ yield of 22 g CH_4_/kg DMI [57, 58]. White box and whisker plots (black symbols in Fig. 3D) represent the Control group; blue boxes (or blue symbols in Fig. 3D) represent the FNZ118-treated animals; purple boxes (or purple symbols in Fig. 3D) represent FNZ142-treated animals; ANOVA *P* value FNZ118 vs. control: ^*^P < 0.05; ANOVA *P*-value FNZ142 vs. control: ^#^*P* < 0.05

CH_4_ emissions from individual animals were measured in respiration chambers over four periods; Round 1 was at 6 weeks of age (pre-weaning), Round 2 at 14 weeks (post-weaning), Round 3 at 9 months (Control and FNZ118 groups only) and Round 4 at 1 year (Fig. 3A and Table 4**)**. CH_4_ production (g/d, Fig. 3A) from the FNZ118 group was significantly lower than Controls at all measurement rounds but CH_4_ yield (g CH_4_/kg DMI/d, Fig. 3B) did not differ from Controls. CH_4_ production from the FNZ142 group was significantly lower than Controls at 6 weeks and 1 year and CH_4_ yield did not differ from Controls at any measurement point. Because there were small differences in animal LWT despite large decreases in DMI in the FNZ118 and FNZ142 groups, CH_4_ production data was also normalised against animal weight (Fig. 3C). The FNZ118 group had lower g CH_4_/kg animal/d at all measurement points, while the FNZ142 group was also lower at 1 year (Fig. 3C). Figure 3D indicates the CH_4_ production from all animals versus DMI from the current study up to 1 year of age as compared to the New Zealand national average of approximately 22 g CH_4_/DMI [57].

CO_2_ measurements followed the same pattern as CH_4_, with CO_2_ production (g/d) in the FNZ118 group lower at all measurement rounds except 9 months, and the FNZ142 group lower at 6 weeks and 1 year compared to the Control group (Table 4). CO_2_ yield (g/kg DMI/d) from the FNZ118 group was higher at 1 year, while the FNZ142 group was higher at 6 weeks compared to the Controls. Hydrogen (H_2_) production (g/d) from both the FNZ118 and FNZ142 groups were lower at 1 year, whereas the yield (g/kg DMI/d) was only lower in the FNZ118 group at 6 weeks of age (Table 4).

### Rumen and faecal pH

To understand the impact of LAB supplementation, the pH of rumen contents and faecal material collected from animals after each measurement round was recorded (Table 5). Mean rumen pH ranged from 5.86–6.01 at 6 weeks (pre-weaning), 5.99–6.32 at 14 weeks (post-weaning), 7.25–7.33 at 9 months and 7.65–7.72 at 1 year. Over the four measurement periods, few differences were detected in the rumen or faecal pH of either the FNZ118 or FNZ142 groups compared to the Control animals.

**Table 5:** Rumen and faecal pH of calves at 6 and 14 weeks, 9 months and 1 year of age^1^

Measurement	Treatment	Week 6	Week 14	9 months	1 year
Rumen pH	Control	5.97 ± 0.11	6.32 ± 0.11	7.31 ± 0.11	7.65 ± 0.11
	FNZ118	5.86 ± 0.11	5.99 ± 0.11^*^	7.25 ± 0.11	7.72 ± 0.11
	FNZ142	6.03 ± 0.11	6.27 ± 0.11	7.33 ± 0.11	7.72 ± 0.11
Faecal pH	Control	7.34 ± 0.13	7.65 ± 0.13	7.15 ± 0.13	7.13 ± 0.13
	FNZ118	7.25 ± 0.13	7.53 ± 0.13	7.21 ± 0.13	7.43 ± 0.13
	FNZ142	7.46 ± 0.13	7.28 ± 0.13^*^	6.88 ± 0.13	7.28 ± 0.13

^1^Numbers are group means ± standard deviation. Mixed effect model post-hoc test of Treatment vs. Control; ^*^P < 0.05

### Volatile and non-volatile fatty acid analyses

**Fig. 4:**
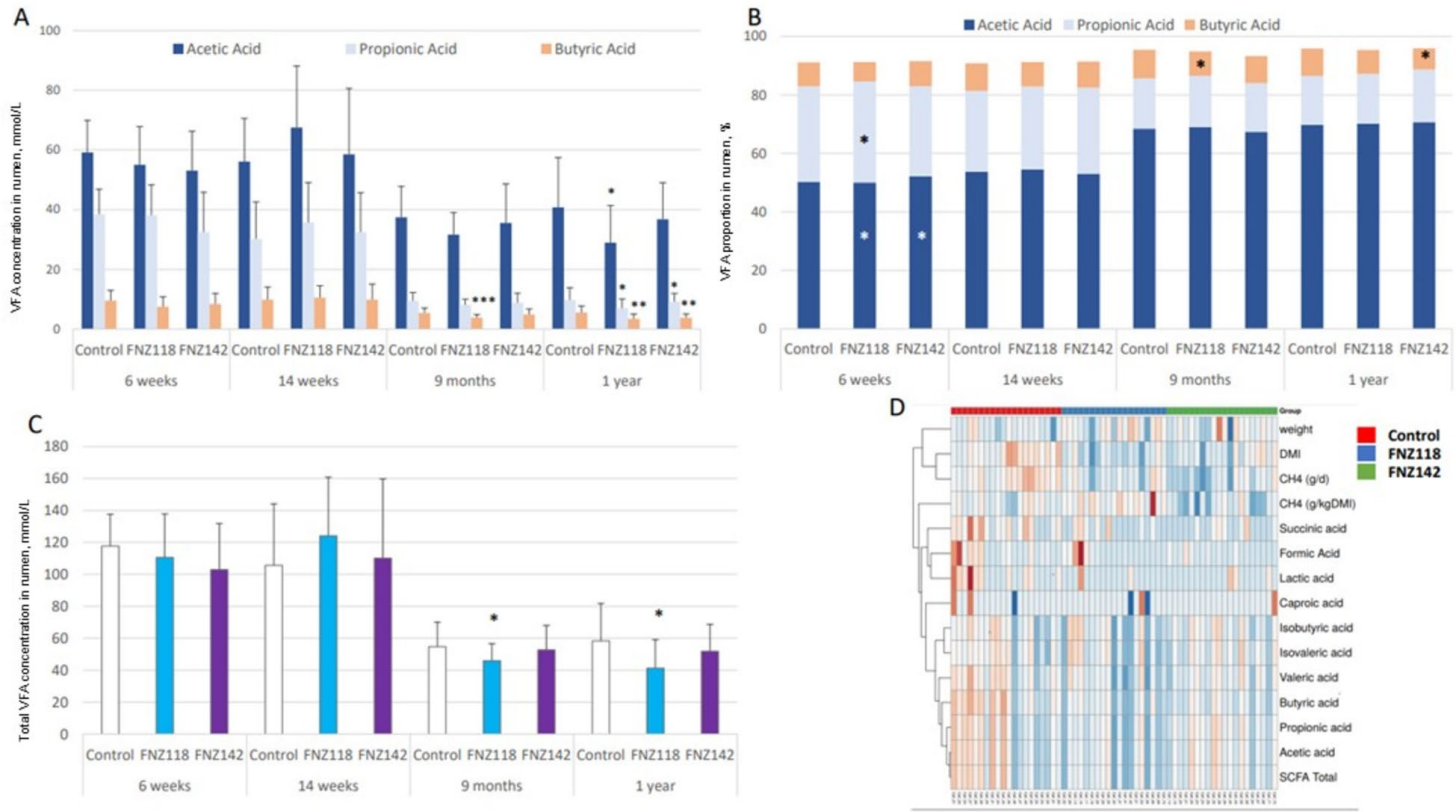
Volatile fatty acids (VFAs) and non-VFAs measured in rumen contents at 6 weeks, 14 weeks, 9 months and 1 year of age. **A** Main VFA concentrations, error bars ± standard deviation. **B** Main VFA proportion. **C** Total VFA and non-VFA concentrations, errors bars ± standard deviation. **D** Heat map generated from DMI, LWT and rumen metabolite data at 1 year of age (Rows are center; unit variance scaling is applied to rows; rows are clustered using Euclidean distance and complete linkage). ANCOMBC *P* value Treatments vs. Control: ^*^P < 0.05; ^**^P < 0.01; ^***^P < 0.001

Rumen VFAs and non-VFAs were measured to understand the physiological impact of supplementation with FNZ118 or FNZ142 on the function of the rumen microbiome. Concentrations of the major ruminal VFAs (acetic acid, propionic acid, and butyric acid) from animals sampled after the four CH_4_ measurement periods are shown in Fig. 4A and Table S9, while minor ruminal VFAs, non-VFAs and formic acid analysed via a derivatisation method, are shown in Table S10. The concentrations of the major VFAs were highest during the first 14 weeks of life (Fig. 4A), when the calves were receiving the FNZ118 or FNZ142 treatments with milk and being offered pelleted feed. Lactic acid concentrations were also highest at these earlier time points (Table S10). After weaning, when the diet contained only pasture, total VFA concentrations decreased in all groups as expected (Fig. 4C). The concentrations of individual VFAs did not differ significantly between the FNZ118 or FNZ142 groups and Controls at either 6 or 14 weeks. After transition onto pasture, butyric acid concentrations were lower in the FNZ118 group at 9 months and acetic acid, propionic acid and butyric acid were lower in these same animals at 1 year (Fig. 4A). The FNZ142 group also showed lower propionic acid and butyric acid at 1 year compared to Controls.

When the major and minor VFAs were expressed as a percentage of total VFAs (Fig. 4B, Table S9) the proportion of acetic acid in the total VFAs increased while the contribution of propionic acid decreased over the four periods of sampling (Table S9 and S10), especially after the shift from pellets onto pasture after 14 weeks. At 6 weeks of age, no major differences in the proportion of VFAs were detected (Fig. 4B). At 9 months and 1 year of age the proportion of butyric acid was lower in the FNZ118 group and lower in the FNZ142 group at 1 year only. The branched chain fatty acid *iso*-butyric acid was present at higher proportions in the 9-month samples of the FNZ118 and FNZ142 groups and the 1-year samples of the FNZ118 group, while *iso*-valeric acid was higher in the FNZ118 and FNZ142 groups at 9 months, and at 1 year in the FNZ118 group, and valeric acid was slightly higher in the FNZ142 group at 9 months (Table S10). Figure 3D shows a heat map using data from the 1-year VFAs, non-VFAs, CH_4_, CH_4_ yield, LWT and DMI measurements. At this time point, the amount of CH_4_ produced per day clustered with DMI and LWT, while CH_4_ yield tended to be associated with VFAs and non-VFAs.

### Rumen and faecal bacterial communities

**Fig. 5:**
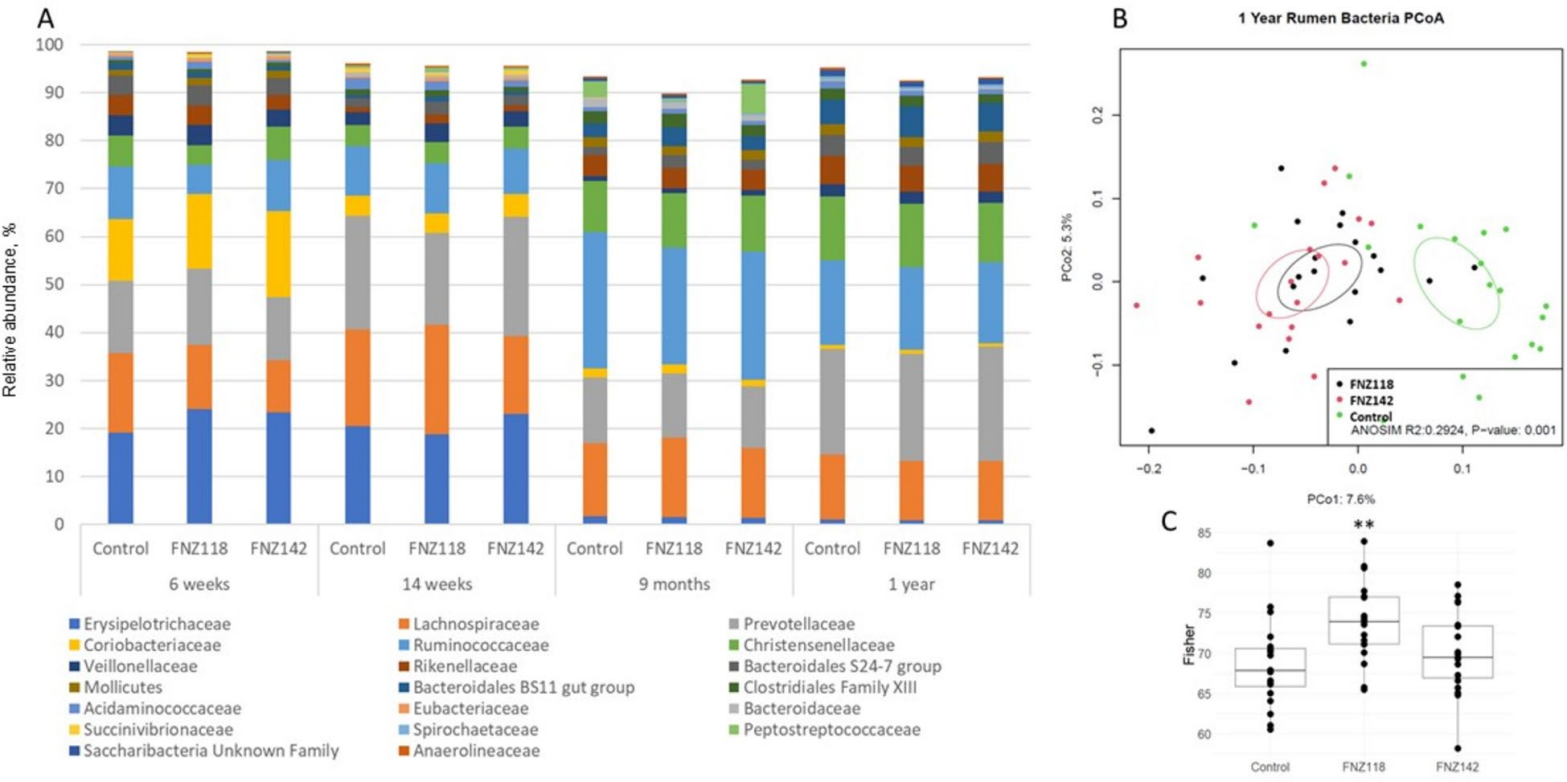
Rumen bacterial community structure. **A** Relative abundances of the top 20 family level bacterial groups identified by OTU analysis in the rumen samples collected at 6 weeks, 14 weeks, 9 months and 1 year of age. **B** PCoA-based on bacterial OTU analysis in the rumen samples collected at 1 year of age. **C** Diversity index analysis of 16S rDNA OTU from rumen samples from 1-year-old heifers. ANOVA *P* value FNZ118 treatment vs. Control: ^**^P < 0.01

Bacterial community analyses were undertaken using 16S ribosomal RNA gene amplicon sequencing to understand the impact of supplementation with FNZ118 or FNZ142 on community composition in the rumen and faeces. Figure 5A and Table S11 show the relative abundances of the top 20 family level groups detected in the rumen. These 20 groups make up 89%–99% of the bacterial population, but only 13 of these groups have cultured representatives. Overall, there were few significant differences in the rumen bacterial community between animals in the FNZ118 or FNZ142 groups and the Controls (Table S11), and differences that were detected affected groups with low relative abundance. Greater differences in species diversity were identified in the 1-year samples as shown by the PCoA analysis (Fig. 5B), where FNZ118 and FNZ142 form a distinct cluster from Control animals (*P* = 0.001). This is supported by a higher Fisher index for the FNZ118 treated calves compared to the Controls (*P* = 0.007) but not for the FNZ142 group (Fig. 5C). Three families (Lachnospiraceae, Prevotellaceae, Ruminococcaceae) were predominant in all samples and together showed > 50% relative abundance at all sampling periods post weaning (Fig. 5A). The Ruminococcaceae and Christensenellaceae families contain many specialised fibre-degrading species and their relative abundances increased following the transition in diet after weaning from milk and pelleted feed to pasture. Members of the Erysipelotrichaceae and Coriobacteriaceae families showed high relative abundances prior to weaning, particularly in the FNZ118 group at 6 weeks of age but then declined after weaning (Fig. 5A). These families contain many lactic acid producers and the relative abundance of the Veillonellaceae family, which contains lactic acid utilizing bacteria, also declined post weaning (Table S11). Although the FNZ118 and FNZ142 groups were fed daily with 5 × 10^10^ CFU for the first 14 weeks, Lactobacillaceae did not feature among the abundant family level groups in the rumen of either the FNZ118 or FNZ142 groups, indicating that these bacteria did not establish populations in the rumen.

The faecal community results are shown in Fig. S2 and Table S12 and demonstrate that the 20 top groups make up 94%–98% of the bacteria in all samples with the two most predominant families being Lachnospiraceae and Ruminococcaceae. Most of the Ruminococcaceae belong to uncultured groups at the genus level. The faecal levels of Lactobacillaceae at 6 weeks (3.2%–3.4% relative abundance) were much higher than detected in the rumen irrespective of whether the animals were receiving the probiotic treatment but declined at 14 weeks and remained at low levels at the 9 month and 1 year samplings. However, when *L. rhamnosus* OTUs were retrieved from the datasets and analysed, the levels detected in the faeces at 6 weeks were significantly higher in the FNZ118 treated calves (0.017% of total OTUs, *P* = 2.5 e-06) than in the Controls (0.0002% of total OTUs), but not at 14 weeks, 9 months or 1 year indicating that feeding FNZ118 had a detectable influence on the calf faecal bacterial community in early gut development.

### Rumen and faecal archaeal communities

**Fig. 6:**
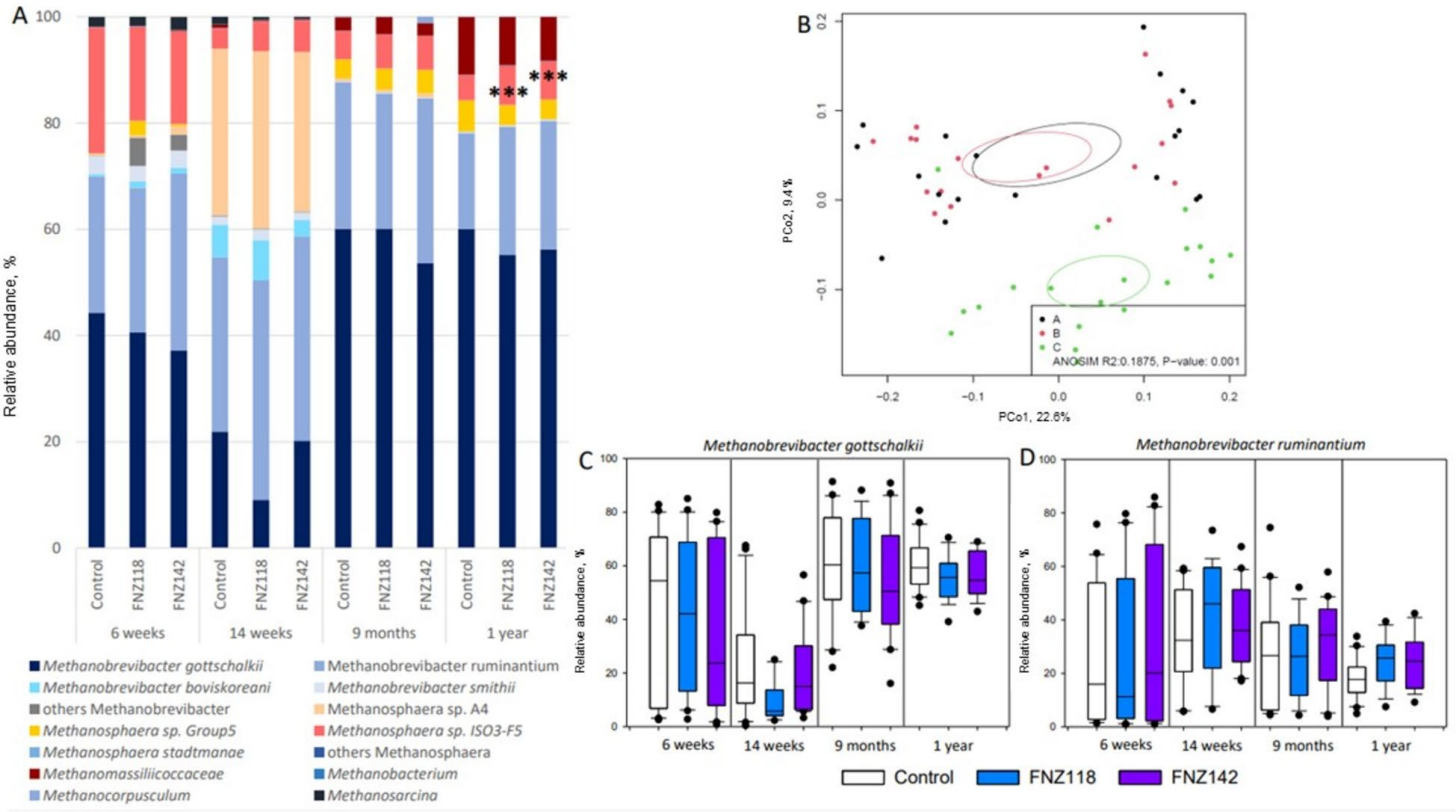
Rumen archaeal community analysis via amplicon sequencing. **A** Relative abundance of the main archaeal groups detected in the rumen samples. **B** PCoA plot based on archaeal OTU relative abundance analysis in the rumen samples collected at 1 year of age. **C** Relative abundance of *M. gottschalkii*. **D** *M. ruminantium* clades from 16S rDNA sequences identified in rumen samples. Box plot data with median and percentile. ^***^P < 0.001

The archaeal communities in the rumen and faeces were also assessed using archaea-specific amplicons. Hydrogenotrophic *Methanobrevibacter* sp. (mainly members of the *M. gottschalkii* and *M. ruminantium* clades) were the dominant methanogens (> 70% relative abundance) in both rumen and faecal samples at 6 weeks, 9 months and 1 year (Fig. 6A, C and D; Tables S13 and S14) and PCoA analysis showed divergence of methanogens between FNZ118 (A) and FNZ142 (B)-treated animals compared to controls (C) (Fig. 6B).

At 14 weeks the relative abundance of *Methanobrevibacter* spp. was slightly lower, principally because of a decline in the abundance of members of the *M. gottschalkii* clade at this time point, with a 44% decrease in its relative abundance in calves receiving FNZ118 compared to Control (Fig. 6C). *Methanobrevibacter* spp. other than members of the *M. gottschalkii* and *M. ruminantium* clades were only apparent in the 6 and 14 week samples. *M. boviskoreani* was the main representative of the *Methanobrevibacter* sp. group detected (Table S13).

Methylotrophic *Methanosphaera* sp. are dependent on methanol for CH_4_ production and showed their highest relative abundances at 6 and 14 weeks (Fig. 6A). However, variation in *Methanosphaera* populations detected in the rumen (Table S13) and faecal samples (Table S14) also indicate that the rumen methanogen community is still being established up to 14 weeks of age. In the 9-month and 1-year samples the relative abundance of *Methanosphaera* sp. was approximately 10%. At the 1 year sampling, the heifers supplemented with FNZ118 or FNZ142 showed significant increases in the relative abundance for *Methanosphaera* sp. ISO3-F5 and Grp 5 in the rumen compared to the Controls (Fig. 6A and Table S13).

Compared with the impact on bacterial diversity (Fig. 5B), pre-weaning administration of FNZ118 or FNZ142 had a larger impact on archaeal diversity in the rumen at 1 year of age (Fig. 6B). However, overall, the rumen and faecal archaeal communities in the treated animals were not significantly different from the Control animals, suggesting that the decreases in CH_4_ emissions were not directly linked to wholesale changes in ruminal or faecal methanogen communities.

Quantitative PCR (qPCR) measurements of total bacteria and total archaea were used to confirm the amplicon sequencing results and demonstrated stable levels during the first year of life (Fig. S3), with no significant effect of either FNZ118 or FNZ142 treatment.

## Discussion

This study demonstrates the efficacy of LAB treatment in early life as a practical means to reduce CH_4_ emissions in ruminant livestock, with an additional beneficial effect on apparent digestive efficiency. This is particularly important for ruminants raised in free-range grazing systems and smallholder farmers in developing countries, where existing CH_4_ mitigation technologies, such as chemical feed additives, are unlikely to be practically applicable or affordable. Providing livestock with carefully selected lactic acid bacteria during this key developmental window has transformative potential, not only in reducing greenhouse gas emissions and mitigating global warming (Sustainable Development Goal (SDG) 13—Climate Action), but also in contributing to closing nutrient deficiency gaps (SDG 2—End Hunger).

Screening a collection of > 1,700 LAB, we were able to identify 2 strains of *L. rhamnosus* that inhibited rumen methanogens and reduced CH_4_ emissions in vitro, and when provided to new-born calves in feed during their first 14 weeks of life, led to sustained reductions in CH_4_ emissions and enhanced feed efficiency through to 1 year of age. The FNZ118-fed group showed reductions in CH_4_ production at all measurement points up to 1 year, where they produced 17% less CH_4_ emissions (g/d) than Control animals. Production of CO_2_ and H_2_ were also lower for the FNZ118 group. The FNZ142-fed group showed a strong, 23% reduction in CH_4_ production at 1 year of age. The reduced CH_4_ emissions in both treatment groups were associated with lower feed intake, with the biggest change observed at 1 year with FNZ118- and FNZ142-fed animals consuming 22% and 16% less pasture on a DMI basis compared to Controls, respectively. Remarkably, the lower DMI of the treatment groups did not affect their LWTs at any timepoint when compared to the Controls. The lower DMI was also consistent with the decreased emissions of H_2_ and CO_2_ and decreased total ruminal VFA concentrations seen in the FNZ118 and FNZ142 groups. These results demonstrate strong reductions in CH_4_ are induced by early life feeding of FNZ118 and FNZ142, which appear to be driven by reduced DMI, but not at the expense of LWT gains. Furthermore, the reductions in CH_4_ production and DMI were surprisingly well sustained, persisting for up to 9 months after ending the treatments.

Regardless of treatment, the heifers in the current study had a lower DMI/kg LWT at 9 and 12 months (0.2 kg DMI/kg LWT excluding the pre-weaned calves) compared to the New Zealand national average for growing dairy heifers (2.5%–3.0% LWT; 0.3 kg DMI/kg LWT). CH_4_ yields in growing dairy heifers globally are reported to be approximately 21–25 g CH_4_/kg DMI [57, 58] similar to the levels observed in the current study (19 g CH_4_/kg DMI). Similarly, the heifers (excluding pre-weaned calves) were producing less CH_4_ per kg DMI than the New Zealand national average of approximately 22 g CH_4_/kg DMI [57, 58].

Studies on mature ruminants using small molecule methanogen inhibitors have shown short-lived reductions in CH_4_ emissions, which resume once treatments are stopped [20, 59–61]. However, there is previous evidence in support of our current study that interventions applied during the early life of developing ruminants can have sustained effects beyond their dosing period. A study in goats demonstrated that microbiome changes detected from early life exposure to a methanogen inhibitor could still be detected three months post-treatment [62]. Furthermore, calves dosed with 3-NOP from birth until 14 weeks of age exhibited reductions in CH_4_ emissions that persisted at similar levels up to at least 1 year of age, but did not show any differences in intake or LWT compared to controls during the pre-weaning period [22]. On the other hand, calves fed a combination of chloroform and 9,10-anthraquinone from 4 d to 12 weeks showed significant decreases in CH_4_ emissions and yields, but these differences lasted for only 2 weeks after dosing ended, and by 24 and 49 weeks their CH_4_ emissions were not different from controls [59]. The treated animals had lower intakes of their pasture mixed ration which carried the treatment doses, but their total intake including concentrate feed during the treatment period, and their pasture intakes at 24 and 49 weeks did not differ from controls. A key difference in this study, compared to the two previously mentioned trials, was that dosing of the methanogen inhibitors started at d 4 rather than from birth and this may explain the short-term effects on CH_4_ observed in that study. This suggests that interventions in a relatively short window immediately after birth may be able to have enduring effects after treatment withdrawal. However, neither of these studies in calves reported a concomitant increase in digestive efficiency [i.e., reduced DMI, maintained LWT] as observed in the current study. Furthermore, the initial reduction in CH_4_ production was less obvious at the later time points.

Differential effects between early and later-life interventions are likely due to development of the gut and establishment of its microbiome as the animal ages. The bovine gut microbiome undergoes substantial changes in microbial composition and diversity over the first two years of life [63]. Aerobic and facultative anaerobic taxa decrease while anaerobic taxa increase over time. Some bacteria essential for mature rumen function, including *Ruminococcus flavefaciens*, *Prevotella ruminicola*,* Selenomonas ruminantium* and *Megasphaera elsdenii* have been detected as early as one day after birth, long before the rumen is active, or ingestion of plant material occurs. This convergence toward a mature microbiome in early life is also observed in the human gut microbiome, suggesting similar forces drive the establishment of gut microbiomes in these two distinct mammalian digestive systems [64]. Early microbial establishment and interactions between the microbiome itself and with the host gut epithelial cells and immune system offer a critical window to shape the developing gut ecosystem and physiology. This presents a valuable opportunity to positively influence the efficiency of ruminant digestion and reduce CH_4_ formation.

In the current study, total H_2_ production was lower or not different in the rumen in calves receiving FNZ118 or FNZ142 in contrast to other studies where large increases were observed [59]. Furthermore, rumen pH was not affected by supplementation of these strains. H_2_ produced from microbial fermentation of feed is the main source of reducing power for CH_4_ production in the rumen. Typically, eliminating CH_4_-producing methanogens, or preventing the formation of CH_4_ through inhibition of key steps in the methanogenesis pathway, results in H_2_ accumulation, which decreases DMI and ultimately milk production and/or LWT gain [65, 66]. Additionally, increased rumen pH levels can also lead to rumen acidosis and rumen tissue damage. Therefore, approaches to reduce CH_4_ that do not increase H_2_ production, such as using LAB, are vital [18, 65] and could be used in a synergistic manner with CH_4_ mitigation options that increase the concentration of H_2_ in the rumen such as 3-NOP. Indeed, nutritional strategies to lower CH_4_ including the use of probiotics like LAB have gained interest due to their ability to stimulate animal productivity, as well as their safety for both young and adult animals. Additionally, they do not negatively impact the nutritional content of the food produced or result in potential food residue issues. While a recent meta-analysis noted that the efficacy of probiotics on CH_4_ emissions is low [29], most studies reported tend to investigate the impacts of probiotics in growing animals (e.g. heifers) or adult cattle rather than early life interventions that target the newborn calf. By using LAB in early life, it appears possible to alter the microbiome of the gastrointestinal tract, thereby favouring factors that result in improved performance and reduced CH_4_ production. While no large differences in the community composition of either the rumen or faeces were observed in the current study, we did not assess changes in other parts of the gastrointestinal tract as the animals were destined for inclusion in a commercial dairy herd. Microbial communities in the entire gastrointestinal tract should be evaluated in future studies.

Most CH_4_ is formed in the rumen and is eructated to the atmosphere via the snout and previous studies showed that low CH_4_ yield sheep had smaller rumen sizes and higher ruminal turnover rates [67]. The smaller rumens with more absorptive rumen surfaces which are thought to select for microorganisms that are capable of fast, heterofermentative growth on soluble sugars, producing less H_2_, leading to less CH_4_ formation [68]. In the current trial, rumen size and feed digestibility were not studied. However, if such changes to rumen size and turnover resulted in more effective forage digestion and absorption, and metabolism of end products, then one would expect the overall feed intake to be lower, as was observed in our study.

The reductions in CH_4_ emissions and ruminal VFA concentrations in animals receiving FNZ118 or FNZ142, did not result in major changes in their microbiome structures. Apart from a transitory decrease in *M. gottschalkii* in the FNZ118 group at 14 weeks, no large differences in the relative abundance of any particular rumen or faecal species attributable to the treatments were identified. The changes that were observed in the microbiome appear to be related to the age and development of the animals and their transition in diet from CMR and pellets onto pasture, rather than being linked to the treatments. It is likely that the main impacts of the *L. rhamnosus* strains occurred in the lower digestive tract. LAB colonise the gut and body cavities of humans and mammals [69–72] and in calves, they may act to create favourable conditions in the hindgut for enhanced digestion and absorption. Frizzo et al. [73] studied the effects of LAB and lactose on calves, and concluded that LAB participated in hindgut colonization processes when fed at an early stage, and improved the establishment of beneficial microbes in the terminal jejunum and ileum. Similarly, bacterial consortia introduced into a rodent model colonized the gut, stimulated the development of the gut wall, influenced exchange between the gut and the mucosa, and aided in immune system maturation [74]. In the current study, the calf faecal community analysis demonstrated that *L. rhamnosus* levels were elevated in the first 6 weeks and thus may have exerted beneficial effects on early hindgut development leading to increased VFA production and absorption in the hindgut and utilisation in the liver. Enhanced VFA supply from the hindgut to the liver could also stimulate vagal nerve afferents to signal the feeding centres in the brain to decrease DMI [53] and thus subsequently reduce CH_4_ emissions through less feed being fermented in the rumen. Between 2% and 12% of ingested energy is lost in the form of eructated CH_4_ [75], and theoretically this energy should be captured in some form if methanogenesis is inhibited. However, studies in which CH_4_ reductions have been reported have generally not demonstrated recovery of this energy as production increases [76].

## Conclusions

The reduced CH_4_ emissions and decreased DMI, coupled with maintenance of LWTs reported here, is one of only a few examples of an intake-sparing effect associated with CH_4_ mitigation and strongly suggests that a change in animal energy metabolism has occurred. This is likely the consequence of better digestive efficiency, through improved digestion and/or nutrient absorption, or via a decrease in animal energy expenditure. However, the mechanism(s) by which feeding FNZ118 or FNZ142 to calves in early life has induced these initial changes and maintained their effects in heifers in the absence of on-going dosing for up to 1 year requires further exploration. While certain *L. rhamnosus* strains have been observed to reduce DMI in other species [77], the mechanisms by which they exert their effect are at present unclear, although of increasing interest, especially in terms of understanding their probiotic efficacy [78]. Experiments investigating whole GI tract digestibility, digesta passage rates, VFA production and absorption rates from the rumen and the hindgut and the metabolic efficiency of FNZ118- and FNZ142-treated heifers are needed to determine the site of action and whether differences in digestive efficiency are involved. Furthermore, the lifetime impacts of these changes should also be investigated, as should impacts on other species of ruminants. Irrespective of the mechanistic details, the potential implications of reducing global ruminant livestock CH_4_ emissions by 17%–23% in the first year of life, while reducing feed intake without impacting calf live weight, warrant further investigation with the aim of determining whether appropriately selected LAB strains could be added to young livestock feed as a means to reduce CH_4_ emissions from ruminants globally.

## Supplementary Information

Additional file 1: Table S1. Microtitre plate setup for the *M. boviskoreani* JH1 growth inhibition assay. Table S2. Microtitre plate setup for the *Methanosphaera* sp. WGK6 growth inhibition assay. Table S3. Microtitre plate setup for the *M. ruminantium* M1 and *M. gottschalkii* D5 growth inhibition assays. Table S4. Ingredients and nutrient composition of pelleted calf feed. Table S5. Quantitative PCR primer pairs targeting rumen methanogen groups. Table S6. Short chain fatty acid concentrations (mmol/L) in RIV assay of *L. rhamnosus* FNZ118 or FNZ142 cells. Table S7. Dietary intakes of calves while in pens at 6 and 14 weeks. Table S8. Live weight and average daily gain of calves prior to chamber measurements at 6 and 14 weeks, 9 months and 1 year of age. Table S9. Major and total volatile fatty acid concentrations in rumen samples. Table S10. Minor volatile fatty acid and non-volatile fatty acid concentrations in rumen samples. Table S11. Relative abundance (± SD) of the top 20 bacterial families detected in the rumen samples. Table S12. Relative abundance (± SD) of the top 20 bacterial families detected in the faecal samples. Table S13. Relative abundance (± SD) of the top 20 archaeal groups detected in the rumen samples. Table S14. Relative abundance (± SD) of the top 20 archaeal groups detected in the faecal samples. Fig. S1. Screening stages to identify LAB strains to test in an early life calf feeding study. Fig. S2. Relative abundance of the top 20 family level bacterial groups identified by OTU analysis in the faecal samples collected from animals at 6 weeks, 14 weeks, 9 months and 1 year of age. Fig. S3. Quantification of 16S rRNA gene copy number per mL of rumen contents for the total archaea and bacteria (A), the main archaeal genera (B), and the main methanogen species (C) in samples collected at 6 weeks, 14 weeks, 9 months and 1 year of age. Supplementary Text on Animal health.

## Data Availability

The datasets generated and/or analysed during the current study will be made available in the Sequence Read Archive (SRA) www.ncbi.nlm.nih.gov/sra/.

## Abbreviations

ADG: Average daily gain
CH_4_: Methane
CMR: Calf milk replacer
CO_2_: Carbon dioxide
DMI: Dry matter intake
DNA: Deoxyribonucleic acid
H_2_: Hydrogen
LAB: Lactic acid bacteria
LWT: Live weight
NZRMMF: New Zealand Ruminant Methane Measurement Facility
OD: Optical density
PCR: Polymerase chain reaction
qPCR: Quantitative PCR
RIV: Rumen in vitro
RNA: Ribonucleic acid
VFAs: Volatile fatty acids
