# Supplementary material for: Early life treatment with Lacticaseibacillus rhamnosus strains drives reduced enteric methane emissions in dairy heifers
Source: J Anim Sci Biotechnol. 2026 Apr 7;17:60. doi: 10.1186/s40104-026-01375-1 (PMC13054989; doi:10.1186/s40104-026-01375-1)
Supplement: Supplementary file 1 — Additional file 1: Table S1. Microtitre plate setup for the M. boviskoreani JH1 growth inhibition assay. Table S2. Microtitre plate setup for the Methanosphaera sp. WGK6 growth inhibition assay. Table S3. Microtitre plate setup for the M. ruminantium M1 and M. gottschalkii D5 growth inhibition assays. Table S4. Ingredients and nutrient composition of pelleted calf feed. Table S5. Quantitative PCR primer pairs targeting rumen methanogen groups. Table S6. Short chain fatty acid concentrations (mmol/L) in RIV assay of L. rhamnosus FNZ118 or FNZ142 cells. Table S7. Dietary intakes of calves while in pens at 6 and 14 weeks. Table S8. Live weight and average daily gain of calves prior to chamber measurements at 6 and 14 weeks, 9 months and 1 year of age. Table S9. Major and total volatile fatty acid concentrations in rumen samples. Table S10. Minor volatile fatty acid and non-volatile fatty acid concentrations in rumen samples. Table S11. Relative abundance (± SD) of the top 20 bacterial families detected in the rumen samples. Table S12. Relative abundance (± SD) of the top 20 bacterial families detected in the faecal samples. Table S13. Relative abundance (± SD) of the top 20 archaeal groups detected in the rumen samples. Table S14. Relative abundance (± SD) of the top 20 archaeal groups detected in the faecal samples. Fig. S1. Screening stages to identify LAB strains to test in an early life calf feeding study. Fig. S2. Relative abundance of the top 20 family level bacterial groups identified by OTU analysis in the faecal samples collected from animals at 6 weeks, 14 weeks, 9 months and 1 year of age. Fig. S3. Quantification of 16S rRNA gene copy number per mL of rumen contents for the total archaea and bacteria (A), the main archaeal genera (B), and the main methanogen species (C) in samples collected at 6 weeks, 14 weeks, 9 months and 1 year of age. Supplementary Text on Animal health. [file 40104_2026_1375_MOESM1_ESM.docx]

## Table S1 Microtitre plate setup for the *M. boviskoreani* JH1 growth inhibition assay

|  | Amount added, μL | | | |
| --- | --- | --- | --- | --- |
| Component | **LAB bacteriocin extract** | **Media control** | **JH1 alone control** | **Nisin** |
| BY medium + formate (3 mol/L) | 150 | 150 | 150 | 150 |
| Phosphate buffer (1 mol/L) | 5 | 90 | 80 | 70 |
| Ethanol (10 mol/L) | 5 | 5 | 5 | 5 |
| Vitamin/CoM solution (1×) | 5 | 5 | 5 | 5 |
| Bacteriocin extract | 75 | 0 | 0 | 0 |
| Nisin (1 mg/mL, 300 μmol/L) | 0 | 0 | 0 | 10 |
| JH1 inoculum | 10 | 0 | 10 | 10 |
| Total | 250 | 250 | 250 | 250 |

## Table S2 Microtitre plate setup for the *Methanosphaera* sp. WGK6 growth inhibition assay

|  | Amount added, mL | | | |
| --- | --- | --- | --- | --- |
| Component | **Media control** | **Nisin control** | **Growth control** | **Bacteriocin test** |
| BRN-10 Media | 3.5 | 3.5 | 3.5 | 3.5 |
| 3M Sodium Formate + 1M Sodium Acetate + 1M Methanol mix | 0.1 | 0.1 | 0.1 | 0.1 |
| 25mg/mL L-cysteine-HCl | 0.1 | 0.1 | 0.1 | 0.1 |
| Methanogen inoculum | 0 | 0.5 | 0.5 | 0.5 |
| Nisin (1 mg/mL, 300 µmol/L) | 0 | 0.2 | 0 | 0 |
| Bacteriocin extract | 0 | 0 | 0 | 0.8 |
| 0.9% NaCl | 0.8 | 0.6 | 0.8 | 0 |
| Additional BRN-10  (in place of inoculum) | 0.5 | 0 | 0 | 0 |
| Total | 5.0 | 5.0 | 5.0 | 5.0 |

## Table S3 Microtitre plate setup for the *M. ruminantium* M1 and *M. gottschalkii* D5 growth inhibition assays

|  | Amount added, mL | | | |
| --- | --- | --- | --- | --- |
| Component | **Media control** | **Nisin** | **Growth control** | **Bacteriocin test** |
| BY medium | 7.0 | 7.0 | 7.0 | 3.5 |
| 3M sodium formate / 1M sodium acetate mix / 1M methanol | 0.2 | 0.2 | 0.2 | 0.1 |
| Vitamin /CoM solution (1×) | 0.2 | 0.2 | 0.2 | 0.1 |
| 25mg/ml L-cysteine-HCl | 0.2 | 0.2 | 0.2 | 0.1 |
| Methanogen inoculum (M1 or D5) | 0 | 1.0 | 1.0 | 0.5 |
| Nisin (1 mg/ mL, 300 µM) | 0 | 0.4 | 0 | 0 |
| Bacteriocin extract | 0 | 0 | 0 | 0.8 |
| Additional BY medium | 1.0 | 0 | 0 | 0 |
| NaCl 0.9% | 1.6 | 1.2 | 1.6 | 0 |
| Total | 10.2 | 10.2 | 10.2 | 5.1 |

**Table S4** Ingredients and nutrient composition of pelleted calf feed

| **Ingredient** | **Inclusion rate, kg** |
| --- | --- |
| Maize | 240 |
| Soya 48 | 200 |
| Barley | 100 |
| Wheat | 80 |
| Molasses | 50 |
| Lucerne, dried | 150 |
| Soya hulls | 150 |
| Limestone | 15 |
| Dicalcium phosphate | 10 |
| Salt | 10 |
| Calf premix + Coccistop^*^ | 2 |
| Mycofix | 1 |
| **Total** | **1008** |
|  |  |
| **Nutrients** | **% Dry matter** |
| Crude protein | 20.3 |
| Neutral detergent fibre | 24.3 |
| Starch | 29.5 |
| Sugar | 7.0 |
| Crude ash | 8.8 |
| Calcium | 1.3 |
| Phosphorus | 0.52 |
| Metabolisable energy (ME) | 12 MJ/kg |

^*^ Decoquinate (Ethyl 6-(decyloxy)-7-ethoxy-4-hydroxyquinoline-3-carboxylate)

## Table S5 Quantitative PCR primer pairs targeting rumen methanogen groups

| **Target group** | **Name** | **DNA sequence (5’→3’)** | **Amplicon, bp** | **Reference** |
| --- | --- | --- | --- | --- |
| Methanomassiliicoccales | Massilo FA1 | GAAGCCCTGGGTCGCAAA | 135 | Adapted from Huang et al. [79] |
|  | Massilo RV1 | TACTCCCCAAGTAGCAGACTT |  |  |
| *Methanobrevibacter* | Mbr FA | CCTCCGCAATGTGAGAAATCGC | 234 | Adapted from Duarte et al. [80] |
|  | Mbr RV | TCACCAGCAATTCCCACAGTT |  |  |
| Total archaea | 915af | AGGAATTGGCGGGGGAGCAC | 491 | Watanabe et al. [81]; Skillman et al. [82] |
|  | 1386r | GCGGTGTGTGCAAGGAGC |  |  |
| Total bacteria | Ba519f | CAGCMGCCGCGGTAANWC | 408 | Lane [83] |
|  | Ba907r | CCGTCAATTCMTTTRAGTTT |  |  |
| *M. gottschalkii* | Gott_F | GGAGATACTATTGGACTTGAGACCG | 223 | This study |
|  | Gott_R | ACTGAAGCAGCTCAAAGCCACCCC |  |  |
| *M. gottschalkii* clade | Gott_Fd | GGAGATACTATTRGACTTGAGACCG | 223 | This study |
|  | Gott_Rd | ACTGRAGCAGCTCAAAGCCACCCC |  |  |
| *M. ruminantium* clade | Rmn_F1 | GTGATACTGTTGAACTTGAGGTC | 119 | This study |
|  | Rmn_R1 | GTCAGGTTCGTTCCAGTTAGC |  |  |
| *M. ruminantium* clade | Rmn_F2 | CGGCTAACTGGAACGAACCTGAC | 305 | This study |
|  | Rmn_R2 | AGTCAAACAAGGTCATCAGCCTGGTA |  |  |
| *Methanosphaera* spp. | Sph_F1 | GAGGTTAGAGGTACTACCGG | 104 | This study |
|  | Sph_R1 | TTCGTTACTCACCGTCAAGAT |  |  |
|  | Sph_R2 | GGAACAACTCGARGCCATTC | 192 | This study |
| *Methanosphaera* spp. | Sph_F2 | GAATGGCYTCGAGTTGTTCC | 198 | This study |
|  | Sph_R3 | CAAATAAGGTCATCAACCTGATTA |  |  |

**Table S6** Short chain fatty acid concentrations (mmol/L) in RIV assay of L. rhamnosus FNZ118 or FNZ142 cells

| **Treatment** | **Incubation time, h** | **Acetic** | **Propionic** | **Butyric** | **Lactic acid** | **Minor SCFAs** | **Total SCFA** |
| --- | --- | --- | --- | --- | --- | --- | --- |
| FNZ118 | 0 | 11.42±0.17 | 2.05±0.03 | 1.41±0.03 | 2.97±0.09 | 0.88±0.03 | 18.73±0.28 |
|  | 2 | 18.83±0.43 | 4.96±0.21 | 2.45±0.05 | 6.28±0.74 | 1.47±0.12 | 33.99±0.26 |
|  | 12 | 39.43±0.46 | 12.13±0.12 | 6.33±0.10 | 0.0±0.0 | 2.25±0.11 | 60.14±0.67 |
|  | 24 | 46.85±2.30 | 14.53±0.68 | 7.74±0.34 | 0.0±0.0 | 2.75±0.12 | 71.87±3.42 |
| FNZ142 | 0 | 11.52±0.14 | 2.07±0.03 | 1.42±0.02 | 3.28±0.31 | 0.93±0.05 | 19.22±0.35 |
|  | 2 | 18.60±0.26 | 4.86±0.16 | 2.41±0.04 | 6.65±0.28 | 2.04±0.48 | 34.57±0.55 |
|  | 12 | 39.06±0.53 | 12.11±0.23 | 6.31±0.15 | 0.0±0.0 | 2.07±0.04 | 59.55±0.85 |
|  | 24 | 48.60±2.37 | 15.19±0.76 | 8.03±0.38 | 0.0±0.0 | 2.79±0.12 | 74.61±3.62 |
| LBF Control | 0 | 11.46±0.16 | 2.06±0.02 | 1.41±0.03 | 2.04±0.53 | 0.89±0021 | 17.86±0.62 |
|  | 2 | 19.08±0.35 | 5.07±0.20 | 2.48±0.04 | 5.97±0.68 | 1.58±0.20 | 34.18±0.95 |
|  | 12 | 37.56±1.62 | 11.52±0.45 | 6.00±0.26 | 0.0±0.0 | 1.98±0.08 | 57.06±2.39 |
|  | 24 | 50.24±1.91 | 15.49±0.55 | 8.19±0.29 | 0.0±0.0 | 2.88±0.11 | 76.80±2.84 |
|  |  |  |  |  |  |  |  |

Numbers indicate SCFA concentrations (mmol/L ± standard error of the mean) in RIV liquid fractions sampled at the indicated time points. The LBF control was *L. bulgaricus* overnight culture filtrate which has no inhibitory effects in RIVs but which accounts for end products from LAB cultures (mainly lactate) which contribute to RIV fermentation. The data was analysed by Student’s *t*-test and there were no significant (*P* < 0.05) differences between any RIV SCFA from FNZ118 or FNZ142 compared to the LBF Control at any time point

**Table S7** Dietary intakes of calves while in pens at 6 and 14 weeks

| **Diet** | **Treatment** | **Week 6^*^** | **Week 14^*^** |
| --- | --- | --- | --- |
|  |  | **Mean** | **Mean** |
| Pellets, kg | Control | 0.682 ± 0.126 | 4.029 ± 0.656 |
|  | FNZ118 | 0.683 ± 0.095 | 3.825 ± 0.280 |
|  | FNZ142 | 0.651 ± 0.139 | 3.887 ± 0.709 |
| Hay, kg | Control | - | 0.330 ± 0.087 |
|  | FNZ118 | - | 0.352 ± 0.076 |
|  | FNZ142 |  | 0.370 ± 0.113 |

^*^ Numbers are weights of feed consumed (kg) per pen of four animals averaged per animal per day, at 6 and 14 weeks of age. 6 L of CMR (in 2 × 3 L feeds am and pm) was consumed by each calf each day at 6 weeks of age, and 0.5 L of CMR per calf each day in the morning only at 14 weeks of age. Hay was not offered to calves until after completion of the Round 1 methane measurement at 6 weeks of age

**Table S8** Live weight and average daily gain of calves prior to chamber measurements at 6 and 14 weeks, 9 months and 1 year of age^1^

| **Measurement** | **Treatment** | **Week 6** | **Week 14** | **9 months** | **1 year** | **REML *P*-values** | | |
| --- | --- | --- | --- | --- | --- | --- | --- | --- |
|  |  |  |  |  |  | **Time** | **Treatment** | **Time:Treatment** |
| Liveweight, kg | Control | 73.20 ± 1.35^d^ | 127.74 ± 2.35^c^ | 223.04 ± 4.26^f^ | 298.7 ± 5.5^a^ | < 2.2e-16 | 0.6774 | 8.72E-10 |
|  | FNZ118 | 72.17 ± 1.33^d^ | 123.66 ± 2.28^bc^ | 239.89 ± 4.58^e^ | 310.08 ± 5.76^a^ |  |  |  |
|  | FNZ142 | 70.17 ± 1.29^d^ | 120.75 ± 2.24^b^ | 236.41 ± 4.51^e^ | 307.14 ± 5.66^a^ |  |  |  |
| Average daily gain, kg | Control | 0.92 ± 0.03^b^ | 0.95 ± 0.03^b^ | 0.60 ± 0.02^d^ | 0.60 ± 0.02^d^ | < 2.2e-16 | 0.05162 | 1.014E-07 |
|  | FNZ118 | 0.88 ± 0.03^b^ | 0.90 ± 0.03^b^ | 0.77 ± 0.02^c^ | 0.67 ± 0.02^a^ |  |  |  |
|  | FNZ142 | 0.88 ± 0.03^b^ | 0.89 ± 0.03^b^ | 0.75 ± 0.02^c^ | 0.66 ± 0.02^a^ |  |  |  |

^1^Numbers are back-transformed group means ± standard error of the mean. Means within a treatment period with different superscripts are significantly different (*P* < 0.05)

**Table S9** Major and total volatile fatty acid concentrations in rumen samples^1^

| **Sampling period** | **Treatment** | **Acetic acid** | **Propionic acid** | **Butyric acid** | **Total VFAs** |
| --- | --- | --- | --- | --- | --- |
| 6 weeks | FNZ118 | 54.69 ± 12.40  (52.27 ± 3.20) | 38.24 ± 10.32  (36.25 ± 3.73) | 7.70 ± 3.55  (7.14 ± 2.47) | 105.33 ± 25.63 |
|  | FNZ142 | 53.42 ± 13.64  (54.78 ± 5.75) | 32.41 ± 13.18  (32.03 ± 6.47) | 8.17 ± 3.42  (8.77 ± 4.37) | 98.46 ± 27.12 |
|  | Control | 59.19 ± 10.73  (52.85 ± 3.82) | 38.45 ± 8.41  (34.25 ± 3.84) | 9.54 ± 3.41  (8.60 ± 3.22) | 112.02 ± 18.73 |
| 14 weeks | FNZ118 | 67.49 ± 20.63  (56.02 ± 5.86) | 35.67 ± 13.42  (29.05 ± 5.30) | 10.52 ± 3.98  (8.63 ± 1.67) | 121.31 ± 36.67 |
|  | FNZ142 | 58.53 ± 22.10  (54.96 ± 5.35) | 32.44 ± 13.25  (30.44 ± 4.28) | 9.87 ± 5.19  (9.22 ± 2.79) | 106.63 ± 39.14 |
|  | Control | 56.12 ± 14.48  (55.79 ± 4.27) | 30.25 ± 12.38  (28.65 ± 5.54) | 9.85 ± 4.20  (9.71 ± 3.10) | 102.28 ± 30.97 |
| 9 months | FNZ118 | 31.65 ± 7.34^*^  (68.95 ± 1.10) | 8.09 ± 1.94  (17.64 ± 1.03) | 3.84 ± 1.01^***^  (8.34 ± 0.79^***^) | 45.93 ± 10.70* |
|  | FNZ142 | 35.58 ± 13.03  (68.04 ± 1.24) | 8.80 ± 3.22  (16.84 ± 0.63) | 4.92 ± 1.81  (9.43 ± 0.82) | 52.28 ± 18.98 |
|  | Control | 37.43 ± 10.36  (68.48 ± 0.88) | 9.43 ± 2.81  (17.16 ± 0.86) | 5.41 ± 1.64  (9.83 ± 0.95) | 54.73 ± 15.30 |
| 1 year | FNZ118 | 28.92 ± 12.48^*^  (70.15 ± 1.70) | 7.02 ± 3.07^*^  (17.06 ± 1.09) | 3.43 ± 1.63^**^  (8.20 ± 1.11^***^) | 41.26 ± 17.85* |
|  | FNZ142 | 36.79 ± 12.22  (70.75 ± 1.26^*^) | 9.23 ± 2.74  (17.95 ± 0.97^***^) | 3.78 ± 1.30^**^  (7.26 ± 0.62^***^) | 51.89 ± 16.77 |
|  | Control | 40.79 ± 16.66  (69.76 ± 1.14) | 9.79 ± 4.06  (16.76 ± 0.83) | 5.51 ± 2.23  (9.40 ± 0.67) | 58.34 ± 23.44 |

^1^Numbers are group means in mM ± standard deviation of individual VFAs as determined by the GC method. Numbers in brackets are % of total VFAs.

T-test probability of Treatment vs. Control; ^*^*P* < 0.05; ^**^*P* < 0.01; ^***^*P* < 0.001

**Table S10** Minor volatile fatty acid and non-volatile fatty acid concentrations in rumen samples^1^

| **Sampling period** | **Treatment** | **Caproic acid** | ***Iso*-butyric acid** | ***Iso*-valeric acid** | **Valeric acid** | **Lactic acid** | **Succinic acid** | **Formic acid** |
| --- | --- | --- | --- | --- | --- | --- | --- | --- |
| 6 weeks | FNZ118 | 0.39 ± 0.01  (0.34 ± 0.01) | 0.54 ± 0.01  (0.53 ± 0.01) | 0.66 ± 0.01  (0.66 ± 0.02) | 3.10 ± 0.08  (2.81 ± 0.05) | 2.73 ± 0.13  (2.47 ± 0.11) | 0.00 ± 0.00  (0.00 ± 0.00) | 0.00 ± 0.00  (0.00 ± 0.00) |
|  | FNZ142 | 0.45 ± 0.02  (0.46 ± 0.02) | 0.47 ± 0.91  (0.48 ± 0.01) | 0.54± 0.02  (0.54 ± 0.01) | 3.00 ± 0.09  (2.94 ± 0.07) | 4.32 ± 0.18  (3.69 ± 0.15) | 0.03 ± 0.01  (0.03 ± 0.01) | 0.02 ± 0.00  (0.03 ± 0.01) |
|  | Control | 0.47 ± 0.02  (0.43 ± 0.02) | 0.52 ± 0.01  (0.47 ± 0.01) | 0.66 ± 0.01  (0.58 ± 0.01) | 3.19 ± 0.06  (2.83 ± 0.05) | 2.83 ± 0.13  (2.61 ± 0.10) | 0.02 ± 0.00  (0.02 ± 0.00) | 0.01 ± 0.00  (0.01 ± 0.00) |
| 14 weeks | FNZ118 | 1.06 ± 0.03  (0.91 ± 0.02) | 0.55 ± 0.02  (0.44 ± 0.01) | 0.71 ± 0.02  0.56 ± 0.01) | 5.30 ± 0.13 (4.38 ± 0.07) | 2.69 ± 0.22  (2.36 ± 0.14) | 0.26 ± 0.01  (0.27 ± 0.01) | 0.06 ± 0.01  (0.06 ± 0.01) |
|  | FNZ142 | 0.76 ± 0.02  (0.71 ± 0.02) | 0.43 ± 0.01  (0.40 ± 0.01) | 0.51 ± 0.01  (0.48 ± 0.01) | 4.09 ± 0.12  (3.78 ± 0.06) | 3.10 ± 0.27  (3.02 ± 0.25) | 0.39 ± 0.02  (0.44 ± 0.02) | 0.05 ± 0.00  (0.05 ± 0.00) |
|  | Control | 0.94 ± 0.02  (0.93 ± 0.02) | 0.46 ± 0.01  (0.46 ± 0.01) | 0.59 ± 0.01  (0.58 ± 0.01) | 4.08 ± 0.08  (3.87 ± 0.04) | 2.57 ± 0.17  (2.52 ± 0.16) | 0.69 ± 0.07  (0.57 ± 0.03) | 0.11 ± 0.01  (0.13 ± 0.01) |
| 9 months | FNZ118 | 0.13 ± 0.00  (0.28 ± 0.00) | 0.77 ± 0.01  (1.66 ± 0.01^***^) | 1.02 ± 0.01  (2.22 ± 0.01^**^) | 0.42 ± 0.00  (0.92 ± 0.00) | 0.21 ± 0.00  (0.47 ± 0.01) | 0.13 ± 0.00  (0.29 ± 0.00) | 0.03 ± 0.00  (0.07 ± 0.00) |
|  | FNZ142 | 0.13 ± 0.00  (0.27 ± 0.00) | 0.97 ± 0.02^*^  (1.84 ± 0.02^***^) | 1.35 ± 0.03^*^  (2.56 ± 0.02^***^) | 0.53 ± 0.01  (1.02 ± 0.01^**^) | 0.20 ± 0.00  (0.46 ± 0.01) | 0.14 ± 0.00  (0.30 ± 0.01) | 0.05 ± 0.00  (0.11 ± 0.00) |
|  | Control | 0.15 ± 0.00  (0.28 ± 0.00) | 0.76 ± 0.01  (1.40 ± 0.01) | 1.06 ± 0.01  (1.96 ± 0.01) | 0.49 ± 0.01  (0.89 ± 0.00) | 0.19 ± 0.00  (0.38 ± 0.01) | 0.18 ± 0.00  (0.35 ± 0.01) | 0.07 ± 0.00  (0.13 ± 0.01) |
| 1 year | FNZ118 | 0.08 ± 0.00  (0.22 ± 0.00) | 0.69 ± 0.02  (1.67 ± 0.01^*^) | 0.80 ± 0.02  (1.93 ± 0.02*) | 0.32 ± 0.01^**^  (0.77 ± 0.01) | 0.03 ± 0.01^*^  (0.08 ± 0.01) | 0.07 ± 0.01^*^  (0.13 ± 0.01^*^) | 0.04 ± 0.00  (0.10 ± 0.01) |
|  | FNZ142 | 0.09 ± 0.00  (0.19 ± 0.00) | 0.74 ± 0.01  (1.43 ± 0.01) | 0.87 ± 0.01  (1.69 ± 0.01) | 0.38 ± 0.01  (0.74 ± 0.00) | 0.04 ± 0.00^*^  (0.09 ± 0.01) | 0.06 ± 0.00^**^  (0.09 ± 0.01^*^) | 0.02 ± 0.00*  (0.05 ± 0.00) |
|  | Control | 0.10 ± 0.00  (0.19 ± 0.00) | 0.78 ± 0.01  (1.42 ± 0.02) | 0.91 ± 0.01  (1.67 ± 0.02) | 0.45 ± 0.01  (0.81 ± 0.01) | 0.14 ± 0.01  (0.20 ± 0.01) | 0.17 ± 0.01  (0.27 ± 0.01) | 0.06 ± 0.00  (0.09 ± 0.01) |

^1^ Numbers are group means in mM ± SEM of individual VFAs. Numbers in brackets are % of total VFAs. Caproic, iso-butyric, iso-valeric and valeric acid were determined by the GC method, while lactic, succinic and formic acid were determined by the SCFA derivatisation method. T-test probability of Treatment vs. Control; ^*^*P*<0.05; ^**^*P* < 0.01

**Table S11** Relative abundance (± SD) of the top 20 bacterial families detected in the rumen samples

|  | **6 weeks** | | | **14 weeks** | | | **9 months** | | | **1 year** | | |
| --- | --- | --- | --- | --- | --- | --- | --- | --- | --- | --- | --- | --- |
| **Order/Family** | **FNZ118** | **FNZ142** | **Control** | **FNZ118** | **FNZ142** | **Control** | **FNZ118** | **FNZ142** | **Control** | **FNZ118** | **FNZ142** | **Control** |
| Erysipelotrichaceae | 24.1 ± 15.5 | 23.3 ± 20.7 | 19.1 ± 12.7 | 18.8 ± 9.2 | 23.0 ± 16.1 | 20.6 ± 17.3 | 1.6 ± 1.4 | 1.4 ± 0.8 | 1.7 ± 1.3 | 0.8 ± 0.2^**^ | 0.9 ± 0.4 | 1.1 ± 0.2 |
| Lachnospiraceae | 13.4 ± 7.3 | 10.9 ± 6.7^*^ | 16.6 ± 8.5 | 22.9 ± 12.5 | 16.3 ± 6.5 | 20.1 ± 11.1 | 16.6 ± 6.9 | 14.5 ± 5.8 | 15.2 ± 6.3 | 13.5 ± 1.3 | 12.4 ± 2.3 | 12.3 ± 2.9 |
| Prevotellaceae | 15.8 ± 8.2 | 13.1 ± 10.6 | 15.1 ± 9.0 | 19.1 ± 9.9 | 24.9 ± 14.3 | 23.8 ± 15.4 | 13.3 ± 9.1 | 12.9 ± 10.8 | 13.8 ± 12.1 | 22.3 ± 5.4 | 23.8 ± 5.2 | 22.1 ± 4.5 |
| Coriobacteriaceae | 15.7 ± 11.5 | 17.9 ± 14.8 | 12.9 ± 11.4 | 4.0 ± 2.6 | 4.6 ± 3.1 | 4.2 ± 2.5 | 1.8 ± 0.9 | 1.3 ± 0.9 | 1.7 ± 1.1 | 0.8 ± 0.2 | 0.7 ± 0.2 | 0.8 ± 0.3 |
| Ruminococcaceae | 6.0 ± 4.1^**^ | 10.7 ± 7.5 | 10.8 ± 6.5 | 10.7 ± 5.1 | 9.6 ± 4.6 | 10.3 ± 4.0 | 24.3 ± 14.9 | 26.8 ± 14.4 | 28.4 ± 15.3 | 17.3 ± 2.4 | 17.1 ± 2.1 | 17.5 ± 2.3 |
| Christensenellaceae | 4.1 ± 3.9 | 7.0 ± 7.5 | 6.5 ± 9.9 | 4.2 ± 1.6 | 4.4 ± 2.8 | 4.5 ± 3.0 | 11.4 ± 4.0 | 11.7 ± 4.0 | 10.8 ± 4.2 | 13.2 ± 3.0 | 12.3 ± 2.9 | 13.4 ± 2.8 |
| Veillonellaceae | 4.3 ± 3.0 | 3.5 ± 2.7 | 4.3 ± 2.1 | 3.9 ± 2.2 | 3.2 ± 2.0 | 2.7 ± 1.7 | 1.1 ± 0.8 | 1.1 ± 1.0 | 1.0 ± 0.9 | 2.6 ± 0.7 | 2.3 ± 0.5^*^ | 2.6 ± 0.4 |
| Rikenellaceae | 4.0 ± 3.4 | 3.1 ± 3.2 | 4.2 ± 3.7 | 2.0 ± 2.7 | 1.2 ± 1.5 | 1.0 ± 1.1 | 4.2 ± 1.9 | 4.3 ± 1.4 | 4.4 ± 1.2 | 5.5 ± 0.9 | 5.9 ± 1.4 | 5.9 ± 1.3 |
| Bacteroidales S24-7 | 4.2 ± 2.5 | 3.4 ± 2.3 | 4.0 ± 2.5 | 2.7 ± 1.5 | 2.3 ± 1.0 | 1.9 ± 1.3 | 2.7 ± 2.3 | 2.0 ± 1.8 | 1.6 ± 1.2 | 3.9 ± 1.0 | 4.5 ± 1.3 | 4.3 ± 0.8 |
| Mollicutes RF9 | 1.6 ± 1.9 | 1.6 ± 1.7 | 1.3 ± 1.9 | 0.006 ± 0.011 | 0.003 ± 0.007 | 0.004 ± 0.006 | 1.8 ± 0.7 | 2.0 ± 0.8 | 2.1 ± 0.6 | 2.1 ± 0.5 | 2.2 ± 0.8 | 2.3 ± 0.6 |
| Bacteroidales BS11 | 1.2 ± 1.0 | 0.9 ± 1.0 | 1.2 ± 1.1 | 0.96 ± 1.38 | 0.73 ± 0.91 | 0.67 ± 0.72 | 4.0 ± 5.6 | 2.8 ± 2.8 | 2.8 ± 2.5 | 6.3 ± 1.8^*^ | 5.8 ± 1.6 | 4.9 ± 1.8 |
| Clostridiales Family XIII | 0.68 ± 0.57 | 0.75 ± 0.77 | 0.76 ± 0.60 | 1.42 ± 0.76 | 0.95 ± 0.44 | 1.12 ± 0.66 | 2.8 ± 1.3 | 2.4 ± 0.7 | 2.5 ± 0.9 | 2.2 ± 0.7 | 2.0 ± 0.5^**^ | 2.5 ± 0.5 |
| Acidaminococcaceae | 1.44 ± 1.61^*^ | 0.59 ± 0.65 | 0.63 ± 0.72 | 1.79 ± 1.16 | 1.41 ± 0.97 | 2.40 ± 2.03 | 1.0 ± 0.7 | 0.8 ± 0.7 | 0.8 ± 0.7 | 1.0 ± 0.5^**^ | 1.0 ± 0.5^**^ | 1.6 ± 0.5 |
| Eubacteriaceae | 0.73 ± 0.51 | 0.66 ± 0.45 | 0.54 ± 0.42 | 0.70 ± 0.44^*^ | 0.47 ± 0.37 | 0.43 ± 0.34 | 0.01 ± 0.02 | 0.01 ± 0.01 | 0.01 ± 0.02 | 0.01 ± 0.01 | 0.01 ± 0.01 | 0.01 ± 0.01 |
| Bacteroidaceae | 0.15 ± 0.22 | 0.30 ± 0.41 | 0.16 ± 0.15 | 0.53 ± 0.79 | 0.73 ± 0.92 | 0.87 ± 1.08 | 1.35 ± 1.09 | 1.25 ± 0.61 | 1.98 ± 1.65 | 0.30 ± 0.08^*^ | 0.41 ± 0.32^*^ | 0.25 ± 0.06 |
| Succinivibrionaceae | 0.57 ± 1.32 | 0.27 ± 0.47 | 0.14 ± 0.14 | 0.76 ± 0.92 | 0.93 ± 1.13 | 0.71 ± 0.80 | 0.07 ± 0.04 | 0.05 ± 0.05 | 0.05 ± 0.04 | 0.08 ± 0.03 | 0.05 ± 0.02 | 0.06 ± 0.03 |
| Spirochaetaceae | 0.09 ± 0.09 | 0.09 ± 0.22 | 0.12 ± 0.22 | 0.35 ± 0.54 | 0.26 ± 0.34 | 0.16 ± 0.21 | 0.23 ± 0.22 | 0.27 ± 0.26 | 0.23 ± 0.25 | 0.42 ± 0.26 | 0.50 ± 0.33 | 0.54 ± 0.28 |
| Peptostreptococcaceae | 0.19 ± 0.39 | 0.20 ± 0.70 | 0.08 ± 0.19 | 0.57 ± 0.66 | 0.22 ± 0.31 | 0.34 ± 0.45 | 0.57 ± 1.01 | 6.28 ± 9.89 | 3.26 ± 7.29 | 0.02 ± 0.01 | 0.02 ± 0.01 | 0.02 ± 0.01 |
| “Saccharibacteria” | 0.02 ± 0.04 | 0.08 ± 0.29 | 0.05 ± 0.14 | 0.04 ± 0.04 | 0.03 ± 0.03 | 0.05 ± 0.09 | 0.71 ± 0.39 | 0.58 ± 0.33 | 0.69 ± 0.45 | 0.99 ± 0.24^**^ | 1.23 ± 0.42 | 1.48 ± 0.51 |
| Bacteroidales RF16 | 0.01 ± 0.01 | 0.02 ± 0.06 | 0.01 ± 0.02 | 0.10 ± 0.22 | 0.07 ± 0.13 | 0.14 ± 0.33 | 0.21 ± 0.26 | 0.27 ± 0.30 | 0.33 ± 0.51 | 0.52 ± 0.33 | 0.68 ± 0.63 | 0.49 ± 0.27 |

T-test probability of Treatment vs. Control, ^*^*P* < 0.05; ^**^*P* < 0.01

**Table S12** Relative abundance (± SD) of the top 20 bacterial families detected in the faecal samples

|  | **6 weeks** | | | **14 weeks** | | | **9 months** | | | **1 year** | | |
| --- | --- | --- | --- | --- | --- | --- | --- | --- | --- | --- | --- | --- |
| **Order/Family** | **FNZ118** | **FNZ142** | **Control** | **FNZ118** | **FNZ142** | **Control** | **FNZ118** | **FNZ142** | **Control** | **FNZ118** | **FNZ142** | **Control** |
| Ruminococcaceae | 24.8 ± 4.8 | 24.2 ± 7.5 | 25.2 ± 5.2 | 46.0 ± 6.0^*^ | 42.0 ± 6.2 | 42.1 ± 4.8 | 40.8 ± 12.2 | 27.7 ± 12.2 | 35.7 ± 13.8 | 43.2 ± 2.0 | 43.0 ± 2.1 | 43.1 ± 2.5 |
| Lachnospiraceae | 23.9 ± 7.0 | 25.0 ± 11.3 | 20.7 ± 4.4 | 17.4 ± 2.9^*^ | 19.1 ± 3.1 | 20.3 ± 3.8 | 10.9 ± 5.2 | 15.4 ± 5.7 | 12.7 ± 6.0 | 7.8 ± 0.8 | 8.2 ± 0.8 | 8.1 ± 0.8 |
| Bacteroidaceae | 8.6 ± 4.8 | 8.8 ± 7.1 | 10.5 ± 4.7 | 4.4 ± 2.0 | 5.4 ± 4.0 | 4.6 ± 1.5 | 2.7 ± 1.7 | 1.3 ± 1.1^**^ | 2.8 ± 2.0 | 3.6 ± 1.2 | 3.7 ± 0.6 | 3.7 ± 0.7 |
| Rikenellaceae | 7.2 ± 5.0 | 6.1 ± 4.5 | 9.1 ± 5.3 | 4.6 ± 2.3 | 7.3 ± 4.0 | 5.4 ± 3.3 | 3.7 ± 1.9 | 3.9 ± 1.4 | 4.5 ± 1.8 | 8.6 ± 1.3 | 8.7 ± 1.5 | 8.0 ± 1.0 |
| Bacteroidales S24-7 | 6.8 ± 2.5 | 5.5 ± 3.4 | 5.9 ± 2.7 | 3.9 ± 3.8 | 3.9 ± 2.5 | 4.5 ± 2.6 | 0.78 ± 0.70 | 2.48 ± 1.93 | 1.47 ± 1.96 | 2.4 ± 0.5 | 2.5 ± 0.6 | 2.4 ± 0.5 |
| Porphyromonadaceae | 3.7 ± 1.4^*^ | 4.8 ± 2.6 | 5.6 ± 2.6 | 0.88 ± 0.37 | 1.10 ± 0.43 | 0.95 ± 0.27 | 0.17 ± 0.14 | 0.10 ± 0.07 | 0.15 ± 0.09 | 0.8 ± 0.2 | 0.7 ± 0.3 | 0.7 ± 0.2 |
| Mollicutes RF9 | 3.2 ± 1.7 | 2.9 ± 3.5 | 4.0 ± 2.0 | 2.5 ± 1.4 | 2.0 ± 1.3 | 2.4 ± 1.3 | 1.5 ± 0.8 | 2.1 ± 0.9 | 1.9 ± 0.6 | 1.8 ± 0.5 | 1.5 ± 0.4 | 1.7 ± 0.4 |
| Erysipelotrichaceae | 6.1 ± 4.3 | 7.0 ± 5.3 | 3.8 ± 2.7 | 2.0 ± 1.4 | 2.0 ± 0.7 | 2.3 ±1.9 | 2.3 ± 1.3 | 2.0 ± 2.7 | 2.1 ± 0.9 | 1.6 ± 0.5 | 1.7 ± 0.5 | 1.5 ± 0.4 |
| Lactobacillaceae | 3.2 ± 2.7 | 3.3 ± 4.8 | 3.4 ± 3.0 | 0.05 ± 0.04 | 0.07 ± 0.05 | 0.06 ± 0.04 | 0.02 ± 0.01 | 0.03 ± 0.02 | 0.03 ± 0.03 | 0.008 ± 0.003 | 0.008 ± 0.004 | 0.006 ± 0.003 |
| Christensenellaceae | 3.5 ± 2.1 | 3.3 ± 1.3 | 3.1 ± 1.3 | 6.9 ± 1.6 | 7.1 ± 2.1 | 7.0 ± 1.4 | 10.2 ± 2.2 | 13.2 ± 3.9^**^ | 9.7 ± 2.3 | 5.2 ± 1.0 | 5.0 ± 0.8 | 5.2 ± 0.8 |
| Peptostreptococcaceae | 2.7 ± 2.9 | 2.5 ± 2.6 | 2.1 ± 1.9 | 0.65 ± 0.94 | 1.27 ± 1.64 | 0.80 ± 0.98 | 10.3 ± 12.9 | 5.0 ± 8.8 | 6.7 ± 9.7 | 2.2 ± 1.8 | 2.5 ± 1.9 | 2.9 ± 2.5 |
| Prevotellaceae | 1.6 ± 1.8 | 2.3 ± 2.8 | 2.0 ± 1.7 | 3.0 ± 1.7 | 2.7 ± 1.8 | 3.2 ± 1.6 | 3.3 ± 6.1 | 9.6 ± 8.1 | 6.9 ± 9.3 | 5.5 ± 1.1 | 5.3 ± 1.4 | 5.0 ± 0.9 |
| Coriobacteriaceae | 1.1 ± 0.9 | 1.5 ± 1.1 | 1.1 ± 2.3 | 0.49 ± 0.65 | 0.33 ± 0.23 | 0.53 ± 0.46 | 2.0 ± 1.3 | 2.0 ± 0.9 | 1.6 ± 0.9 | 0.20 ± 0.12 | 0.25 ± 0.15 | 0.18 ± 0.07 |
| Veillonellaceae | 0.48 ± 0.26 | 0.49 ± 0.27 | 0.70 ± 0.48 | 0.79 ± 1.10 | 0.63 ± 0.45 | 0.94 ± 0.99 | 0.38 ± 0.95 | 0.83 ± 0.66 | 0.75 ± 1.26 | 3.0 ± 0.3 | 3.1 ± 0.2 | 3.1 ± 0.3 |
| Acidaminococcaceae | 0.52 ± 0.20 | 0.39 ± 0.20 | 0.52 ± 0.26 | 0.53 ± 0.21 | 0.57 ± 0.20 | 0.63 ± 0.25 | 0.18 ± 0.22 | 0.78 ± 0.63 | 0.42 ± 0.58 | 0.43 ± 0.11 | 0.48 ± 0.11 | 0.46 ± 0.14 |
| Clostridiales Family XIII | 0.44 ± 0.25 | 0.34 ± 0.24 | 0.42 ± 0.22 | 1.06 ± 0.40 | 0.94 ± 0.46 | 0.97 ± 0.27 | 3.2 ± 1.1 | 3.3 ± 1.8 | 2.7 ± 1.1 | 3.45 ± 0.60 | 3.34 ± 0.55 | 3.56 ± 0.61 |
| Peptococcaceae | 0.11 ± 0.11 | 0.08 ± 0.06 | 0.09 ± 0.05 | 0.15 ± 0.08 | 0.19 ± 0.17 | 0.17 ± 0.10 | 0.57 ± 0.25^*^ | 0.27 ± 0.28 | 0.40 ± 0.27 | 0.47 ± 0.18 | 0.48 ± 0.20 | 0.48 ± 0.12 |
| Bacteroidales RF16 | 0.07 ± 0.12 | 0.03 ± 0.06 | 0.07 ± 0.11 | 0.48 ± 0.88 | 0.21 ± 0.37 | 0.24 ± 0.37 | 0.21 ± 0.26 | 0.18 ± 0.15 | 0.20 ± 0.19 | 1.7 ± 0.5 | 1.9 ± 0.7 | 1.8 ± 0.5 |
| Bacteroidales BS11 | 0.03 ± 0.02 | 0.01 ± 0.01 | 0.02 ± 0.01 | 0.04 ± 0.11 | 0.02 ± 0.01 | 0.02 ± 0.02 | 0.74 ± 1.29 | 3.46 ± 2.82 | 2.00 ± 2.97 | 1.3 ± 0.4 | 1.1 ± 0.4 | 1.2 ± 0.3 |
| Bacteroidales Incertae Sedis | 0.00 | 0.00 | 0.00 | 0.10 ± 0.24 | 0.04 ± 0.07 | 0.05 ± 0.06 | 0.81 ± 0.69 | 0.36 ± 0.56^*^ | 0.90 ± 0.86 | 2.0 ± 0.5 | 1.9 ± 0.7 | 1.7 ± 0.6 |

T-test probability of Treatment vs. Control, ^*^*P* < 0.05; ^**^ *P*< 0.01

**Table S13** Relative abundance (± SD) of the top 20 archaeal groups detected in the rumen samples

|  | **6 Weeks** | | | **14 Weeks** | | | **9 Months** | | | **1 Year** | | |
| --- | --- | --- | --- | --- | --- | --- | --- | --- | --- | --- | --- | --- |
| **Clade/Genus** | **FNZ118** | **FNZ142** | **Control** | **FNZ118** | **FNZ142** | **Control** | **FNZ118** | **FNZ142** | **Control** | **FNZ118** | **FNZ142** | **Control** |
| *Mbb.* *gottschalkii* clade | 40.6 ± 28.8 | 37.3 ± 31.8 | 44.3 ± 28.8 | 9.1 ± 7.5^*##^ | 20.2 ± 16.0 | 21.9 ± 19.6 | 60.1 ± 16.8 | 53.7 ± 20.6 | 60.1 ± 19.7 | 55.2 ± 8.5 | 56.2 ± 8.5 | 60.1 ± 9.5 |
| *Mbb.* *ruminantium* clade | 27.2 ± 29.0 | 33.3 ± 33.6 | 25.7 ± 23.5 | 41.3 ± 21.3 | 38.5 ± 15.0 | 32.8 ± 17.9 | 25.5 ± 14.7 | 31.0 ± 15.7 | 27.6 ± 19.9 | 24.1 ± 9.6* | 24.2 ± 10.4* | 18.1 ± 7.3 |
| *Methanosphaera* sp. ISO3-F5 | 17.7 ± 7.0 | 17.5 ± 10.1 | 23.7 ± 10.4 | 5.6 ±- 7.6 | 5.9 ± 6.2 | 3.8 ± 5.1 | 6.5 ± 3.7 | 6.4 ± 3.9 | 5.4 ± 3.0 | 7.4 ± 2.2*** | 7.2 ± 2.5** | 4.78 ± 1.3 |
| *Methanosphaera* sp. Grp 5 | 2.8 ± 8.5 | 0.38 ± 1.6 | 0.02 ± 0.02 | 0.02 ± 0.06 | 0.02 ± 0.02* | 0.01 ± 0.01 | 4.0 ± 3.5 | 4.5 ± 3.2 | 3.6 ± 2.3 | 3.8 ± 1.4** | 3.7 ± 1.8** | 5.9 ± 2.7 |
| Methanomassiliicoccales Grp 10 sp. | 0.00 | 0.18 ± 0.80 | 0.02 ± 0.01 | 0.04 ± 0.15 | 0.02 ± 0.06 | 0.01 ± 0.03 | 2.5 ± 2.6 | 1.7 ± 1.9 | 1.8 ± 2.5 | 7.6 ± 3.2 | 6.4 ± 4.7 | 8.2 ± 3.7 |
| *Methanosphaera* sp. A4 | 0.43 ± 0.15 | 1.6 ± 5.5 | 0.49 ± 0.29 | 33.3 ± 17.2 | 30.0 ± 15.2 | 31.4 ± 12.9 | 0.50 ± 0.47 | 0.74 ± 0.71 | 0.54 ± 0.40 | 0.08 ± 0.10 | 0.06 ± 0.05 | 0.03 ± 0.05 |
| Methanomassiliicoccales Grp 9 sp. ISO4-G1 | 0.01 ± 0.01 | 0.00 | 0.00 | 0.07 ± 0.19 | 0.13 ± 0.26 | 0.57 ± 1.5 | 0.34 ± 0.67 | 0.22 ± 0.31 | 0.29 ± 0.56 | 0.53 ± 0.65* | 0.69 ± 1.13 | 1.04 ± 0.74 |
| Methanomassiliicoccales Grp 4 sp. MpT1 | 0.00 | 0.00 | 0.00 | 0.00 | 0.00 | 0.00 | 0.19 ± 0.37 | 0.13 ± 0.16 | 0.20 ± 0.43 | 0.34 ± 0.32 | 0.59 ± 0.77 | 0.54 ± 0.51 |
| *Methanobrevibacter smithii* | 2.9 ± 2.4 | 3.3 ± 2.7 | 3.3 ± 2.8 | 2.0 ± 5.6 | 1.4 ± 1.3 | 1.6 ± 1.3 | 0.16 ± 0.08 | 0.17 ± 0.09 | 0.15 ± 0.07 | 0.22 ± 0.07 | 0.28 ± 0.13 | 0.24 ± 0.08 |
| Methanomassiliicoccales Grp 12 sp. ISO4-H5 | 0.00 | 0.00 | 0.00 | 0.00 | 0.00 | 0.00 | 0.08 ± 0.15 | 0.06 ± 0.10 | 0.10 ± 0.27 | 0.12 ± 0.09* | 0.16 ± 0.18 | 0.26 ± 0.21 |
| Methanomassiliicoccales Gp 8 sp. WGK1 | 0.00 | 0.00 | 0.00 | 0.00 | 0.00 | 0.00 | 0.06 ± 0.15 | 0.04 ± 0.07 | 0.07 ± 0.12 | 0.36 ± 0.25 | 0.22 ± 0.30* | 0.60 ± 0.66 |
| *Methanocorpusculum* sp. | 0.00 | 0.00 | 0.00 | 0.00 | 0.00 | 0.00 | 0.00 | 1.13 ± 2.99 | 0.06 ± 0.20 | 0.00 | 0.00 | 0.00 |
| Methanomassiliicoccales Grp 3b sp. | 0.00 | 0.00 | 0.00 | 0.00 | 0.00 | 0.00 | 0.02 ± 0.03 | 0.17 ± 0.43 | 0.03 ± 0.03 | 0.08 ± 0.05 | 0.10 ± 0.13 | 0.12 ± 0.08 |
| Methanomassiliicoccales Grp 3a sp. | 0.00 | 0.00 | 0.00 | 0.00 | 0.00 | 0.00 | 0.00 | 0.04 ± 0.11 | 0.00 | 0.05 ± 0.04 | 0.05 ± 0.05 | 0.06 ± 0.05 |
| *Methanosphaera cuniculi* | 0.02 ± 0.01 | 0.01 ± 0.02 | 0.02 ± 0.01 | 0.06 ± 0.05 | 0.06 ± 0.04 | 0.06 ± 0.03 | 0.02 ± 0.02 | 0.03 ± 0.03 | 0.02 ± 0.02 | 0.13 ± 0.10* | 0.11 ± 0.15 | 0.07 ± 0.08 |
| *Mbb.* *arboriphilus* clade | 0.02 ± 0.02 | 0.01 ± 0.02 | 0.02 ± 0.01 | 0.02 ± 0.02 | 0.03 ± 0.02 | 0.03 ± 0.03 | 0.02 ± 0.02 | 0.01 ± 0.01 | 0.02 ± 0.02 | 0.01 ± 0.01 | 0.01 ± 0.02 | 0.01 ± 0.01 |
| *Mbb.* *boviskoreani* clade | 6.5 ± 2.0 | 4.0 ± 3.0 | 0.45 ± 1.3 | 7.6 ± 14.1 | 3.0 ± 5.7 | 6.1 ± 9.7 | 0.03 ± 0.06 | 0.00 | 0.00 | 0.00 | 0.00 | 0.01 ± 0.02 |
| *Methanobacterium alkaliphilum* | 0.00 | 0.00 | 0.00 | 0.00 | 0.00 | 0.00 | 0.02 ± 0.06 | 0.00 | 0.00 | 0.00 | 0.00 | 0.01 ± 0.02 |
| *Methanosphaera stadtmanae* | 0.09 ± 0.14 | 0.04 ± 0.04 | 0.05 ± 0.04 | 0.05 ± 0.03 | 0.04 ± 0.03 | 0.05 ± 0.03 | 0.00 | 0.00 | 0.00 | 0.02 ± 0.02 | 0.02 ± 0.02 | 0.02 ± 0.02 |
| *Mbb. acididurans* | 0.00 | 0.00 | 0.00 | 0.00 | 0.00 | 0.00 | 0.00 | 0.01 ± 0.02 | 0.00 | 0.00 | 0.00 | 0.01 ± 0.01 |

T-test probability of Treatment vs. Control, ^*^*P* < 0.05; ^**^*P* < 0.01; ^***^*P* < 0.001. T-test probability of FNZ118 vs. FNZ142, ^#^*P* < 0.05; ^##^*P* < 0.01

**Table S14** Relative abundance (± SD) of the top 20 archaeal groups detected in the faecal samples

|  | **6 weeks** | | | **14 weeks** | | | **9 months** | | | **1 year** | | |
| --- | --- | --- | --- | --- | --- | --- | --- | --- | --- | --- | --- | --- |
| **Clade/Genus** | **FNZ118** | **FNZ142** | **Control** | **FNZ118** | **FNZ142** | **Control** | **FNZ118** | **FNZ142** | **Control** | **FNZ118** | **FNZ142** | **Control** |
| *Mbb.* *gottschalkii* clade | 39.3 ± 32.2 | 40.1 ± 32.7 | 39.4 ± 30.2 | 10.0 ± 11.6 | 17.6 ± 18.2 | 14.8 ± 14.8 | 33.2 ± 18.4 | 32.4 ± 19.7 | 37.9 ± 19.3 | 25.9 ± 12.7 | 25.3 ± 10.4 | 24.5 ± 9.4 |
| *Mbb.* *ruminantium* clade | 30.0 ± 34.9 | 31.8 ± 36.1 | 32.3 ± 32.2 | 29.3 ± 20.9 | 23.5 ± 14.4 | 23.6 ± 16.6 | 52.8 ± 18.0 | 51.5 ± 17.4 | 49.5 ± 19.9 | 46.3 ± 18.9 | 54.9 ± 12.0 | 47.8 ± 11.4 |
| *Methanosphaera* sp. ISO3-F5 | 17.2 ± 13.9 | 16.4 ± 7.3 | 19.8 ± 11.8 | 3.2 ± 5.4 | 2.2 ± 2.1 | 1.4 ± 1.1 | 4.8 ± 2.3 | 5.6 ± 3.1 | 5.8 ± 2.9 | 5.5 ± 3.3 | 5.3 ± 2.2 | 5.1 ± 2.0 |
| *Methanosphaera* sp. Grp 5 | 0.96 ± 2.9 | 0.01 ± 0.04 | 0.01 ± 0.01 | 0.00 | 0.00 | 0.00 | 6.4 ± 3.4 | 4.2 ± 2.0 | 4.6 ± 3.5 | 4.3 ± 2.3 | 4.3 ± 2.3 | 5.8 ± 3.5 |
| Methanomassiliicoccales Grp 10 sp. | 0.00 | 0.00 | 0.00 | 0.00 | 0.00 | 0.00 | 0.20 ± 0.71 | 1.1 ± 1.0 | 0.56 ± 1.03 | 0.05 ± 0.09 | 0.03 ± 0.03 | 0.04 ± 0.05 |
| *Methanosphaera* sp. A4 | 0.94 ± 0.46 | 0.78 ± 0.50 | 0.89 ± 0.48 | 45.7 ± 19.9 | 48.1 ± 19.2 | 48.7 ± 14.5 | 0.88 ± 0.60 | 0.73 ± 0.56 | 1.10 ± 0.96 | 0.02 ± 0.02 | 0.05 ± 0.05 | 0.04 ± 0.05 |
| Methanomassiliicoccales Grp 9 sp. ISO4-G1 | 0.00 | 0.00 | 0.00 | 0.00 | 0.00 | 0.00 | 0.02 ± 0.06 | 0.12 ± 0.16 | 0.12 ± 0.23 | 0.01 ± 0.03 | 0.00 | 0.01 ± 0.04 |
| Methanomassiliicoccales Grp 4 sp. MpT1 | 0.00 | 0.00 | 0.00 | 0.00 | 0.00 | 0.00 | 0.01 ± 0.03^*^ | 0.07 ± 0.08 | 0.05 ± 0.06 | 0.93 ± 2.5 | 0.40 ± 0.55 | 0.58 ± 0.77 |
| *Mbb. smithii* | 1.3 ± 1.8 | 1.9 ± 1.6 | 6.6 ± 14.5 | 0.46 ± 0.63 | 0.62 ± 0,60 | 0.54 ± 0.49 | 0.18 ± 0.08 | 0.14 ± 0.07 | 0.15 ± 0.09 | 0.17 ± 0.11 | 0.19 ± 0.08 | 0.17 ± 0.07 |
| Methanomassiliicoccales Grp 12 sp. ISO4-H5 | 0.00 | 0.00 | 0.00 | 0.00 | 0.00 | 0.00 | 0.01 ± 0.02 | 0.02 ± 0.03 | 0.02 ± 0.03 | 0.02 ± 0.03 | 0.05 ± 0.10 | 0.03 ± 0.06 |
| Methanomassiliicoccales Grp 8 sp. WGK1 | 0.00 | 0.00 | 0.00 | 0.00 | 0.00 | 0.00 | 0.02 ± 0.07 | 0.07 ± 0.10^*^ | 0.01 ± 0.02 | 0.16 ± 0.27 | 0.14 ± 0.20 | 0.14 ± 0.19 |
| *Methanocorpusculum* sp. | 0.00 | 0.00 | 0.00 | 0.00 | 0.00 | 0.00 | 1.39 ± 3.9 | 3.87 ± 11.7 | 0.06 ± 0.19 | 12.7 ± 15.5 | 5.2 ± 7.7 | 11.1 ± 14.6 |
| Methanomassiliicoccales Grp 3b sp. | 0.00 | 0.00 | 0.00 | 0.00 | 0.00 | 0.00 | 0.05 ± 0.09 | 0.10 ± 0.31 | 0.07 ± 0.17 | 1.3 ± 2.2 | 1.8 ± 2.9 | 1.8 ± 2.3 |
| Methanomassiliicoccales Grp 3a sp. | 0.00 | 0.00 | 0.00 | 0.00 | 0.00 | 0.00 | 0.01 ± 0.03 | 0.02 ± 0.08 | 0.01 ± 0.04 | 2.4 ± 4.0 | 2.0 ± 2.4 | 2.7 ± 1.7 |
| *Methanosphaera cuniculi* | 0.01 ± 0.01 | 0.01 ± 0.01 | 0.00 | 0.00 | 0.00 | 0.00 | 0.04 ± 0.02 | 0.02 ± 0.03 | 0.04 ± 0.04 | 0.04 ± 0.04 | 0.08 ± 0.10 | 0.06 ± 0.07 |
| *Mbb.* *arboriphilus* clade | 0.00 | 0.01 ± 0.01 | 0.01 ± 0.02 | 0.02 ± 0.02 | 0.01 ± 0.02 | 0.02 ± 0.02 | 0.01 ± 0.01 | 0.02 ±- 0.02 | 0.02 ± 0.01 | 0.01 ± 0.01 | 0.02 ± 0.02 | 0.02 ± 0.02 |
| *Mbb.* *boviskoreani* clade | 10.0 ± 24.4 | 8.8 ± 23.7 | 0.92 ± 1.3 | 11.3 ± 16.8 | 7.8 ± 12.0 | 10.9 ± 16.2 | 0.00 | 0.03 ± 0.07 | 0.00 | 0.00 | 0.00 | 0.00 |
| *Methanobacterium alkaliphilum* | 0.00 | 0.00 | 0.00 | 0.00 | 0.00 | 0.00 | 0.03 ± 0.09 | 0.00 | 0.02 ± 0.07 | 0.01 ± 0.01 | 0.00 | 0.00 |
| *Methanosphaera stadtmanae* | 0.04 ± 0.03 | 0.03 ± 0.03 | 0.03 ± 0.02 | 0.03 ± 0.02 | 0.03 ± 0.02 | 0.03 ± 0.02 | 0.01 ± 0.01^*^ | 0.01 ± 0.01 | 0.00 | 0.02 ± 0.02 | 0.01 ± 0.02 | 0.02 ± 0.02 |
| *Mbb. acididurans* | 0.01 ± 0.02 | 0.01 ± 0.01 | 0.01 ± 0.02 | 0.00 | 0.00 | 0.00 | 0.01 ± 0.03 | 0.01 ± 0.02 | 0.01 ± 0.02 | 0.01 ± 0.01 | 0.01 ± 0.01 | 0.01 ± 0.03 |

T-test probability of Treatment vs. Control, ^*^*P* < 0.05


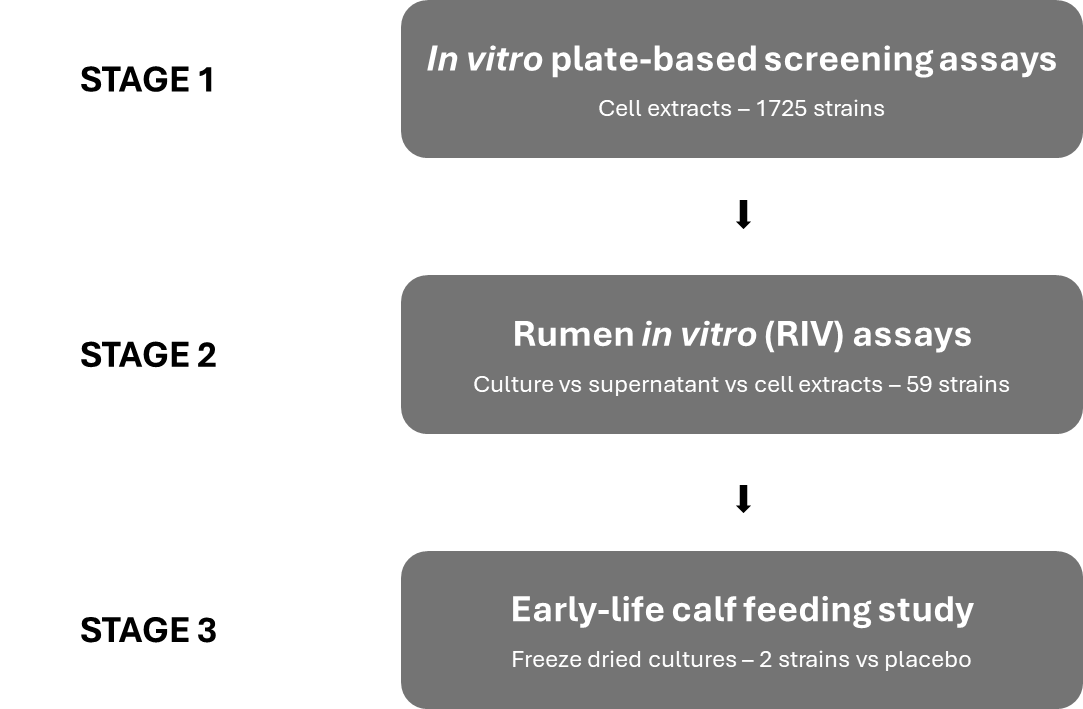


**Fig. S1** Screening stages to identify LAB strains to test in an early life calf feeding study


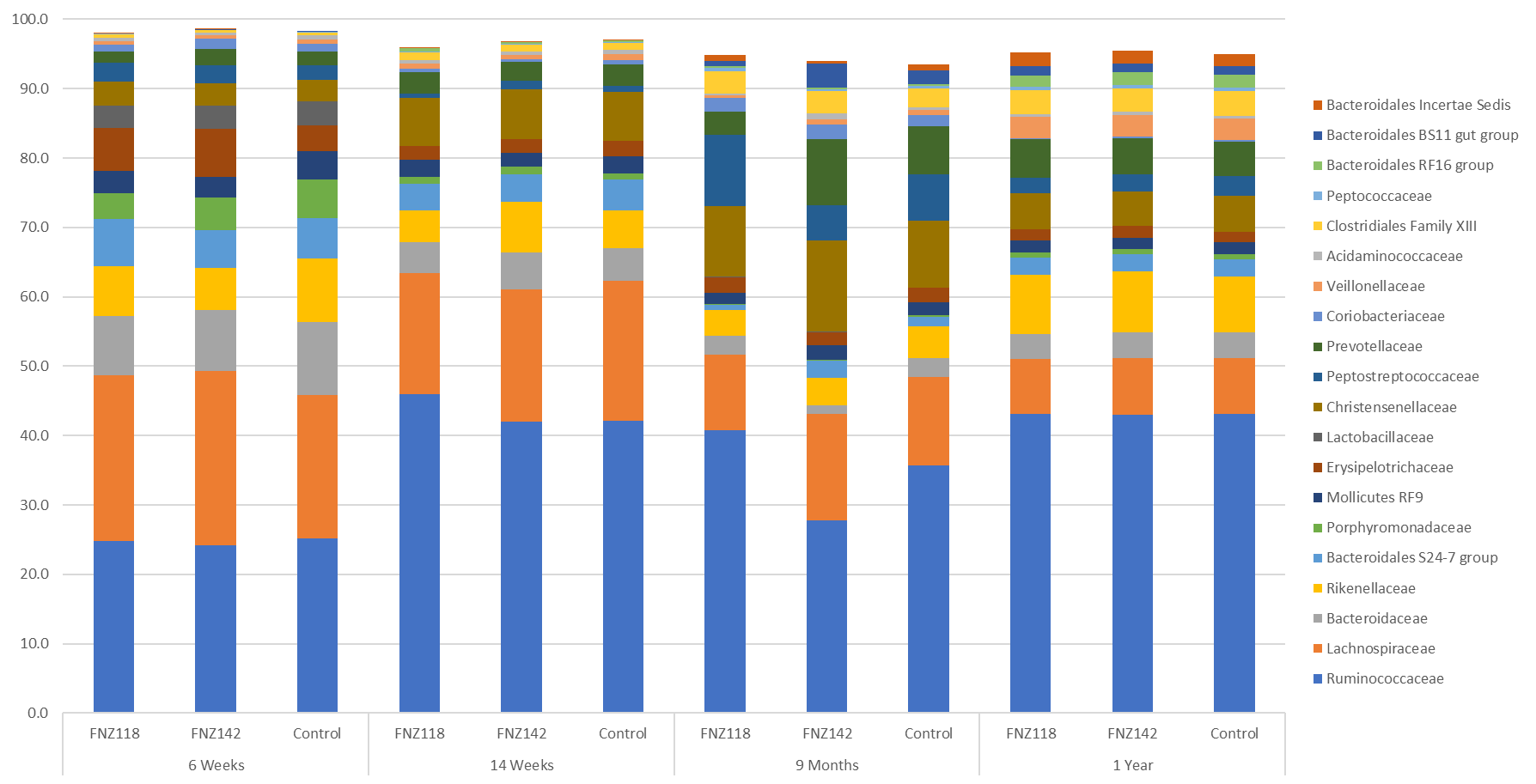


**Fig. S2** Relative abundance of the top 20 family level bacterial groups identified by OTU analysis in the faecal samples collected from animals at 6 weeks, 14 weeks, 9 months and 1 year of age

**
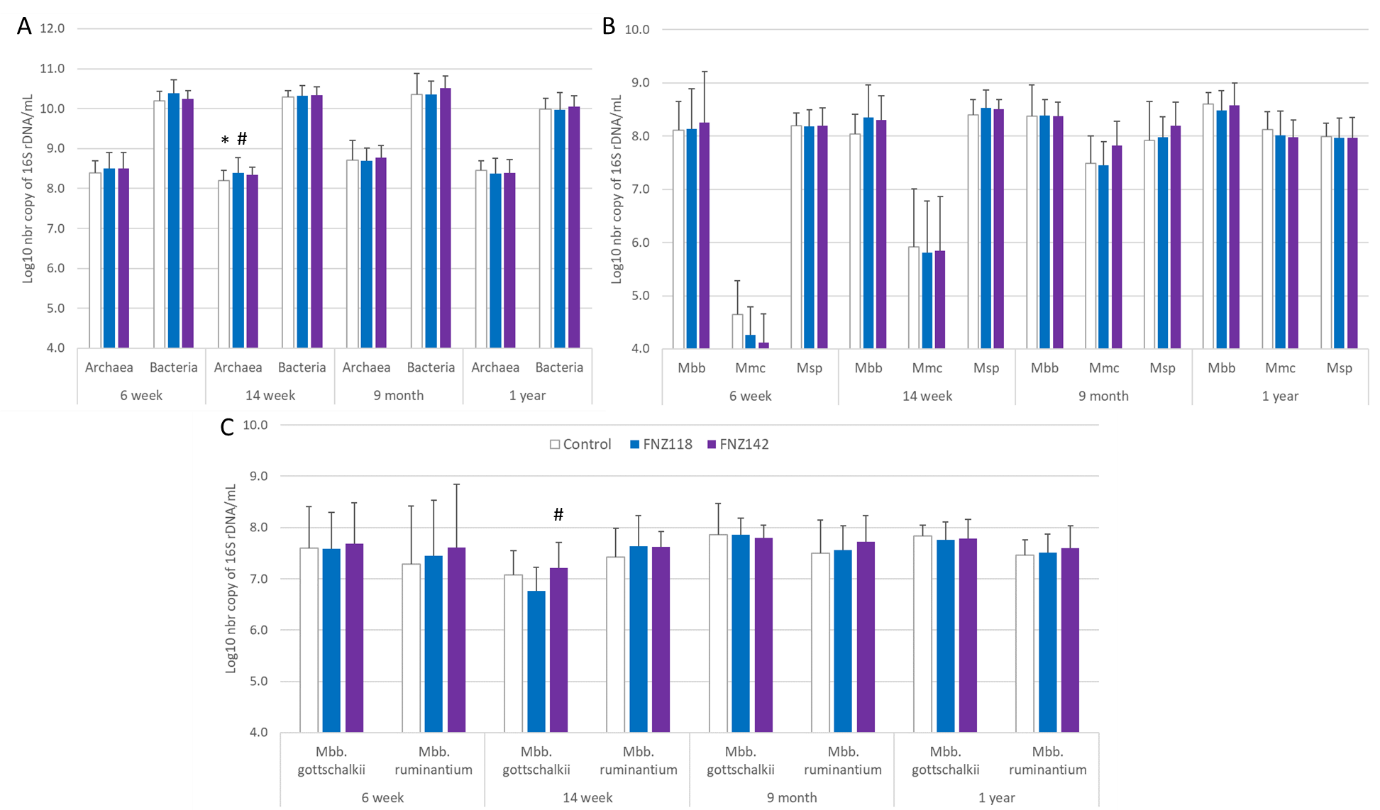
**

**Fig. S3** Quantification of 16S rRNA gene copy number per mL of rumen contents for the total archaea and bacteria (A), the main archaeal genera (B), and the main methanogen species (C) in samples collected at 6 weeks, 14 weeks, 9 months and 1 year of age. M, *Methanobrevibacter*; Msp, *Methanosphaera*; Mmc, Methanomassiliicoccales. ANOVA *P* value FNZ118 vs. Control: ^*^*P* < 0.05; FNZ142 vs. Control: ^#^*P* < 0.05

**Supplementary References**

79. Huang XD, Martinez-Fernandez G, Padmanabha J, Long R, Denman SE, McSweeney CS. Methanogen diversity in indigenous and introduced ruminant species on the Tibetan Plateau. Archaea. 2016;2016:5916067.

80. Duarte A, Durmic Z, Vercoe PE, Chaves AV. Dose-response effects of dietary pequi oil on fermentation characteristics and microbial population using a rumen simulation technique (Rusitec). Anaerobe. 2017;48:59-65.

81. Watanabe T, Asakawa S, Nakamura A, Nagaoka K, Kimura M. DGGE method for analyzing 16S rDNA of methanogenic archaeal community in paddy field soil. FEMS Microbiol Lett. 2004;232:153-163.

82. Skillman LC, Evans PN, Naylor GE, Morvan B, Jarvis GN, Joblin KN. 16S ribosomal DNA-directed PCR primers for ruminal methanogens and identification of methanogens colonising young lambs. Anaerobe. 2004;10:277-285.

83. Lane DJ. 16S/23S rRNA Sequencing. In: Stackebrandt E, Goodfellow M, editors. Nucleic Acid Techniques in Bacterial Systematic. New York: John Wiley and Sons; 1991. p. 115-175.

**Supplementary Text**

Animal health

Calves were disbudded and received vaccinations and anti-parasitic treatments according to the Massey University Dairy 4 farm animal health and welfare protocols. Calf disbudding was carried out by an MFS veterinarian at 10-14 days of age, under sedation (with the use of xylazine, local anaesthetic, and nonsteroidal anti-inflammatory drug, Metacam). Vaccinations given were for Clostridia, *Salmonella*, leptospirosis, and Bovine Viral Diarrhoea (BVD) and were provided under the direction of the MFS veterinarian. Scouring calves were assessed with clinical diagnostics tests and where necessary calves were isolated and given intravenous electrolyte therapy. Calf navels were checked daily by calf rearing staff at feeding and navel spray was applied twice daily until the navel was dry. MFS veterinarian examination of navels was carried out at disbudding while the calves were under sedation and any infected navels were assessed and treated by an MFS veterinarian.

After returning to Massey University Farms and being adapted to full pasture grazing, several animals began to show clinical signs of coccidiosis in mid-December 2021. One animal was given oral Baycox (45 mL), subcutaneous Metacam, and 3-4 L electrolytes, with vitamins B1 and B12 and housed at the veterinary clinic for 3 days to recover. Two other animals showed clinical signs of coccidiosis and were treated orally with Baycox (45 mL), and as a precaution the remaining animals were also treated. After heavy rainfall events in December 2021 and February 2022, a number of the heifers displayed signs of lameness, and between-claw footrot was diagnosed. There were 23 cases reported within the 72 animals originally enrolled in the study and these cases were treated with intracillin (17-20 mL per heifer depending on LWT). After the Round 3 CH_4_ measurement a quarantine drench (Zolvix Plus, Elanco NZ, 0.1 mL/kg LW; active ingredients: 25 mg/mL monepantel, 2 mg/mL abamectin) was given to each heifer before being returned to the Massey University farms.
